# Monitoring of biofouling communities in a Portuguese port using a combined morphological and metabarcoding approach

**DOI:** 10.1038/s41598-020-70307-4

**Published:** 2020-08-10

**Authors:** Joana Azevedo, Jorge T. Antunes, André M. Machado, Vitor Vasconcelos, Pedro N. Leão, Elsa Froufe

**Affiliations:** 1 Interdisciplinary Centre of Marine and Environmental Research, CIIMAR/CIMAR, Matosinhos, Portugal; 2 Faculty of Sciences, University of Porto, Porto, Portugal

**Keywords:** Computational biology and bioinformatics, Environmental sciences

## Abstract

Marine biofouling remains an unsolved problem with a serious economic impact on several marine associated industries and constitutes a major vector for the spread of non-indigenous species (NIS). The implementation of biofouling monitoring programs allows for better fouling management and also for the early identification of NIS. However, few monitoring studies have used recent methods, such as metabarcoding, that can significantly enhance the detection of those species. Here, we employed monthly monitoring of biofouling growth on stainless steel plates in the Atlantic Port of Leixões (Northern Portugal), over one year to test the effect of commercial anti-corrosion paint in the communities. Fouling organisms were identified by combining morpho-taxonomy identification with community DNA metabarcoding using multiple markers (16S rRNA, 18S rRNA, 23S rRNA, and COI genes). The dominant colonizers found at this location were hard foulers, namely barnacles and mussels, while other groups of organisms such as cnidarians, bryozoans, and ascidians were also abundant. Regarding the temporal dynamics of the fouling communities, there was a progressive increase in the colonization of cyanobacteria, green algae, and red algae during the sampled period with the replacement of less abundant groups. The tested anticorrosion paint demonstrated to have a significant prevention effect against the biofouling community resulting in a biomass reduction. Our study also reports, for the first time, 29 NIS in this port, substantiating the need for the implementation of recurring biofouling monitoring programs in ports and harbours.

## Introduction

Marine biofouling organisms colonize immersed surfaces and form communities on diverse biogenic habitats^1,2^. This colonization process is most intense in coastal or shallow waters, where species diversity, temperatures, nutrient levels, and availability of submerged substrata are usually higher compared to offshore areas^3^. Biofouling represents a major economic issue for maritime industries and it raises important environmental concerns due to increased drag, exhaust emissions, and operational costs^4,5^ while creating also the potential for invasion by non-indigenous species (NIS)^6–8^. Stainless steel surfaces are frequently used as a material in vessels, port installation surfaces, cooling water circuits, ships or related equipment, and are known to be extensively colonized by fouling organisms when submerged in water^9,10^. Biofouling monitoring programs are therefore essential to help in the understanding and mitigation of such negative impacts. Until recently, the diversity of biofouling organisms has been mainly assessed through morphological identification^11^. However, the implementation of methods that allow for earlier and more comprehensive detection of the micro- and macro-fouler colonizers is desirable^12,13^. Metabarcoding approaches are useful to validate species identification based on morphological traits, with the possibility of “sight-unseen” detection of target species^14,15^, and detecting of species with a high resolution while processing all the specimens from a sample simultaneously^16^. Moreover, metabarcoding approaches can simultaneously employ different gene-markers to minimize taxonomic bias^17,18^, which is very useful to accurately characterize global routes of indigenous and non-indigenous species^8,19^. Such strategies have recently been successfully applied to unravel the composition and structure of eukaryotic macrofouling assemblages^20–22^, to detect introduced species^23–26^ and to perform port biodiversity surveys^27,28^. Regarding the latter, only a few studies looked into the effect of antifouling or anticorrosion paints^22,29,30^.

Biofouling monitoring programs are still seldom used in Portuguese ports. This is particularly relevant considering that the “European Marine Strategy Framework Directive”^31^ requires solid knowledge about the presence and distribution of NIS to achieve accurate environmental status^32^. Furthermore, the International Convention for the Control and Management of Ships' Ballast Water and Sediments (Ballast Water Management Convention or BWM Convention), adopted in 2004 by the International Maritime Organization^33^, which aims to reduce the spread and the new introduction of harmful organisms, requires the monitoring of invasive species transported by ships. The BWM Convention has developed a sampling protocol for fouling organisms that has been used to perform a Port Biological Baseline Survey (PBBS) and is capable of supporting NIS management strategies^34,35^. Notwithstanding, the Portuguese list of NIS reflects a clear lack of information about northern Portuguese ports, encompassing only species (133 marine and brackish) monitored in central or southern estuaries (i.e. Ria de Aveiro, Sado, Guadiana, Ria Formosa) and the archipelagos (Madeira and Azores)^36^.

Taking this into consideration, this study aimed to assess the colonization of biofouling species (indigenous and NIS) in the cruise-ship terminal marina of the Port of Leixões, Northern Portugal, on a monthly basis and over one year. The Port of Leixões is the second most important Portuguese port in terms of volume of shipping and marine traffic, and its traffic is steadily increasing every year, representing around 25% of the Portuguese international trade^37^. The settlement of fouling communities and the presence of NIS in the port water were assessed and the colonization of surfaces covered with and without anti-corrosion paint was compared. The temporal succession of the fouling taxa colonizing steel plates was evaluated with a complementary metabarcoding and morphological assessment. Our results show that the employed four gene marker combination, together with the morphological identification is a powerful approach to accurately assess the biofouling diversity since several phyla/taxa were only retrieved with a particular marker. Additionally, this study revealed for the first time a list of 29 possible non-indigenous species present in Northern Portuguese waters.

## Materials and methods

### Study site and experiment set-up

**Figure 1:**
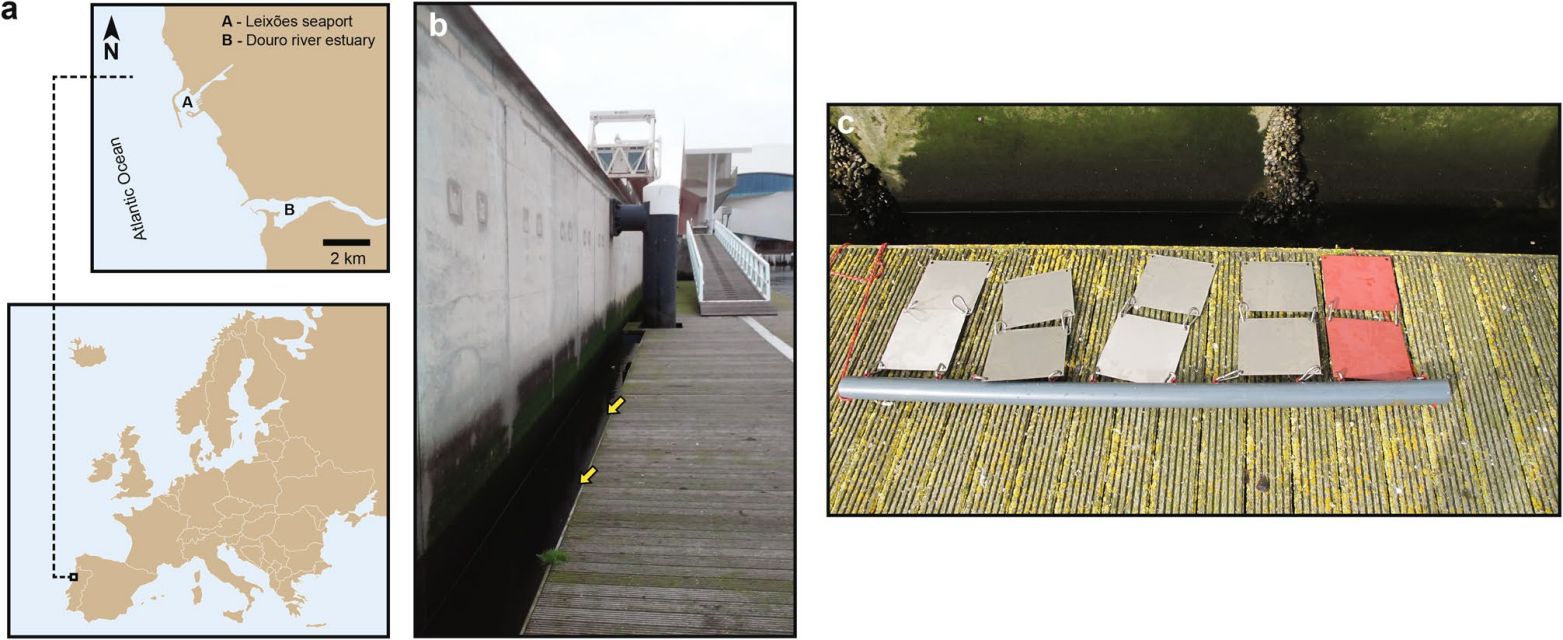
Sampling site: (**a**) Location of the Cruise Terminal of the Port of Leixões in Northern Portugal, Europe; (**b**) Marina floating platform; (**c**) Biofouling monitoring structure composed of ten stainless-steel plates.

The analysis of the biofouling colonization on the immersed steel panels was conducted in the cruise terminal of the Port of Leixões (northern Portugal) (41°10′39.32″ N, 8°42′8.78″ W). At the Port of Leixões, a pier that docks both large cruise passenger ships and freighter ships was selected as the study site (Fig. 1). We followed the PBBS survey and used already existing structures in the port (i.e., a pier in which we installed our monitoring structure) to implement a more suitable sampling method.

The experimental design for the detection of biofoulers was selected according to a previous guideline^38^, the “Standard Test Method for Testing Anti-fouling Panels in Shallow Submergence”^39^ (ASTM D3626-78-a, 2012) and to a previous empirical study^40^. The PBBS survey suggests that while working with PVC plates, these should be sanded before the deployment to provide a more hospitable substrate for organisms. As we were working with steel plates, we did not sand the plates beforehand. Besides this consideration, we followed most of the recommendations advised by the guide for PVC plates as they are similar to previous studies that employed steel surfaces^41,42^.

The employed artificial structure was constituted by 10 stainless-steel (316 2B) square panels (20 cm × 20 cm × 2 mm), attached by carabineers and supported by a grey PVC tube (Fig. 1c). To test the effect of anti-corrosion paint in the communities, two plates were covered with anti-corrosion paint (painted panels) and the remaining eight plates were left untreated (bare panels). The painted panels were professionally covered with a non-biocidal anti-corrosion paint (Intersheen 579, International Paint Ltd, Gateshead, UK) by the Port Authority of Douro, Leixões and Viana do Castelo (APDL) personnel. The panels were vertically submerged in two five-panel rows (each containing one painted and four bare panels) at depths of between 1.00 m and 1.30 m (due to the floating system of the platform) and placed within 10 cm of each other (to avoid contact between the panels). The plates were positioned with a Northeast (NE)/Southwest (SW) orientation (the Northeast-facing side was more exposed to sunlight).

### Temporal sample collection

To determine the temporal succession of the attached communities, on each month, randomly selected 16 cm^2^ grids were sampled from the Northeast-facing side of (i) the two painted panels (n = 3 for each panel), and (ii) four random bare steel panels (n = 3 for each panel). The selected grids were scraped using a sterile stainless-steel surgical blade and the same grid was not scraped in the subsequent months. The sampling was conducted monthly from April 2016 until March 2017. All panels were permanently immersed throughout the sampling campaign, only re-surfaced for the sampling of the selected grids as detailed above and were then vertically submerged with the same orientation. A total of 216 grids were scraped throughout the one-year sampling period (i.e. 6 painted panel grids and 12 bare panel grids pre month). The scraped biomass was placed into sterile five-millilitre microcentrifuge tubes, placed on ice coolers, and transported into the laboratory. In the last sampling month, the bare panel labelled as P6, was fully scraped on both Southwest- and Northeast-facing sides (n = 2) making a total of 218 scraped samples. Aliquots of the scraped samples were used for morpho-taxonomic analysis and the remaining biomass was lyophilized and stored at − 80 °C until DNA extraction procedures (See Table S1 for a summary of the samples surveyed and sequenced). All panels were photographed monthly for subsequent image analyses during the 12-month sampling period.

### Morpho-taxonomic analysis by microscopy

To visually identify biofouling species, a qualitative analysis of the samples was performed under a light microscope at × 10, × 40, and × 100 magnifications. Photographs were obtained using a digital camera unit Olympus U-TV1X-2 DP72 attached to an Olympus SZX10 microscope or an Olympus BX41 phase-contrast microscope. Identification was performed to the lowest possible taxonomic level, by consulting taxonomic guides^43,44^, and the World Register of Marine Species (WORMS)^45^ and AlgaeBase^46^ databases.

### Monitoring of biofouling colonization using ImageJ software

The biofouling colonization was evaluated by photographing all panels in both facing sides using a digital camera (Canon PowerShot G11). To quantitatively determine the colonization percentage of the panels, the image processing package Fiji which includes the ImageJ software v.1.46 was used^47,48^. The obtained three-dimensional images were converted to integrate single two-dimensional (2D) images through the process of “Maximum Intensity Projection (MIP)”. The biofouling coverage was calculated using the MIP 2D by converting the images to 8-bit grey images, which were then processed into binary images through the “process-make binary” command. Noisy signals were removed using the “Filters” option and biofouling colonization percentage was calculated using the “Analyze particles” option.

### Biofouling biomass per area determination

Biofouling biomass was calculated by weighing individually wet-panels every month in a stainless-steel digital scale (Küchewaage PC-KW 1061, Germany) at the study site before each sampling, by always taking into account the preservation of the biofouling cover. The weight of the panels was corrected by subtracting the corresponding non-fouled panel weight and divided by the panel area. The area where the carabineers attached to the panel (4 × 16 cm^2^) were not considered, resulting in a total area of 336 cm^2^.

### DNA extraction, gene marker selection, library preparation, and sequencing

Lyophilized material from each scraped grid samples (n = 216) was homogenized using a bleached pestle and a mortar. The dry weight (20 to 250 mg) was then transferred to PowerSoil DNA Isolation Kit PowerBead tubes (MO BIO Laboratories Inc., USA). Additionally, six additional samples (three from each side) of the fully scraped bare panel from the last sampling month were subjected to the same DNA extraction protocol. All the material used in the weighing was previously sterilized by submerging it on a 10% bleach solution for 10 min and thoroughly rinsing it with ultrapure water before being used. To achieve physical lysis of cell material, samples were submitted to two cycles of 5,600 g for 15 s in a PreCellys®24 homogenizer (Bertin Technologies, France) and, subsequently, DNA was extracted, according to the kit manufacturer’s instructions, making a total of 222 DNA extracts. Then, DNA extracts from each panel were pooled (n = 3) to yield one painted and one bare panel sample per month. Lastly, DNA extracts were scraped from two whole bare plates, one from plates facing Northeast and other from plates facing Southwest (See Table S1). With this, a total of 26 different DNA extract pools were obtained*.* DNA purity and quantification for these samples were performed using a multi-mode microplate reader (BioTek® Synergy™ HT, USA) and confirmed by visualization on a 1% agarose (w/v) gel. Table S1 provides a summary of the sampling procedure leading to the DNA samples that were submitted for sequencing.

To select the different molecular markers to be used, preliminary polymerase chain reaction (PCR) runs were performed using one protocol (see below) and adjusting annealing temperatures as needed, for each gene marker region. Amplicons derived from the 16S rRNA, 18S rRNA, 23S rRNA, and the mitochondrial cytochrome oxidase subunit I (COI) genes were selected for the metabarcoding approach (Table S2). Individual samples from 10 main taxonomic biofouling groups (i.e. green, brown and red algae; poriferans, bryozoans, ascidians, cnidarians, annelids, molluscs, and crustaceans) that had previously been collected in the same sampling location were used as mock communities and DNA extraction was conducted as mentioned above. The primers used for the amplification of 16S rRNA (341F-785R)^49^, 18S rRNA (TAReuk454FWD1- TAReukREV3)^50^, COI (mlCOIintF^51^- dgHCO2198^52^) and 23S rRNA (p23SrVF- p23SrVR)^53^ genes were selected to amplify the main fouling groups (see Table S2). The PCR conditions (25 μL reactions) were as follows: each reaction contained 2.5 μL 10 × Invitrogen PCR Buffer, 0.5 μL 10 mmol of each primer, 1.5 μL 50 mmol MgCl_2_, 0.5 μL 10 mmol dNTPs, 0.1 μL Invitrogen Taq DNA Polymerase and approximately 2 μL DNA template. Thermal cycling involved an initial denaturation at 94 °C for 3 min; denaturation at 94 °C (30 s); annealing at 70 °C (2 min) for 16S rRNA, 70 °C (1 min) for 18S rRNA, 62 °C (1 min) for 23S rRNA, and 62 °C (30 s) for COI. The extension step was conducted at 72 °C (1 min) repeated for 40 cycles and a final extension at 72 °C for 10 min. DNA amplification for each of the gene markers was confirmed by 1% agarose (w/v) gel electrophoresis. All PCR reactions included negative and positive controls; the positive controls were samples from mock communities and composed of fouling target taxa chosen for each of the gene markers (see Table S2). This was conducted in order to optimize the PCR steps for each of the markers (verified by the presence of a single PCR band in the 1% agarose gel (w/v)) and to allow subsequent sequencing. The 26 DNA samples (Table S1) were then shipped to LGC Genomics GmbH (Berlin, Germany), where the libraries were prepared. Products from three independent PCR runs for each sample (n = 26) were pooled in equal volumes and purified using the PCR Clean-Up System (Promega, Madison, DE, USA). Equimolar aliquots of all samples were combined for each of the markers, and for PCR-amplified DNA fragments, per sample, two distinct PCR reactions were performed. Subsequently, the sequencing was carried out with 300 bp paired-end reads using the Illumina MiSeq platform (MiSeq Reagent Kit v3) to obtain MiSeq—Illumina libraries. All primers used for PCR amplification are depicted in Table S2. Sequence reads for this study were submitted to the European Nucleotide Archive (ENA) and were made available under the accession number PRJEB33602.

### Bioinformatics analysis and operational taxonomic units (OTUs) identification

The bioinformatics analyses were subdivided in three main stages, pre-processing and clean-up of raw datasets, OTU clustering, and OTU identification. The pre-processing was conducted at LGC genomics, and initially, all raw reads (in FASTQ format) were subjected to base calling and demultiplexing pre-processing (Illumina bcl2fastq v.1.8.4). After that, the forward and reverse sequences were merged using BBMerge (v.34.48).

#### 16S rRNA and 18S rRNA genes

The clean-up of 16S and 18S rRNA gene sequences was done with the Quantitative Insights Into Microbial Ecology (QIIME, v.1.8.0) software^54^ (using default settings). All reads containing less than 100 bp of length and a phred quality score of < Q20^55^ were discarded. Chimeric sequences were identified and removed using UCHIME v.4.2.40. At the end of this clean-up stage, taxonomic unit (OTU) picking was performed by using UCLUST v1.2.22q^56^ within QIIME using the closed-reference method. The canonical clustering threshold of 97% for 16S rRNA was adopted^57^, and the same threshold was made for other markers following recent metabarcoding studies that tried to identify non-indigenous species in marine fouling communities^18^. To classify the 16S and 18S rRNA reads, Greengenes (release gg_13_5)^58^ and SILVA (release 108)^59^ were used, respectively, as implemented in the QIIME pipeline and using the default QIIME-based wrapper classifier, the RDP naive Bayesian classifier^60^.

#### 23S rRNA and COI genes

The clean-up and clustering of 23S rRNA and COI gene marker sequences were carried out using the USEARCH sequence analysis tool (v.8.1.1831), namely the read quality filtering and fastq_filter scripts to remove reads containing less than 100 bp of length and phred quality score of < Q20. Next, the UCHIME v.4.2.40 software was used to remove chimeric sequences. The classification of OTUs and taxonomy assignment of both the 23S rRNA and COI gene marker was carried out as follows: 1) all OTU sequences were searched against the NT-NCBI database (downloaded on 25/11/2018)) using the BLASTn algorithm (v.2.5.0) (NT-NCBI Parameters—-db nt -remote -max_target_seqs 5 -outfmt '6 qseqid sseqid length pident gaps sstart send qcovs evalue bitscore staxids stitle'); 2) OTUs without a hit in the NT-NCBI database were blasted against the BOLD System databases^61^ (BOLD Parameters -max_target_seqs 5 -num_threads 20 -outfmt '6 qseqid sseqid length pident gaps sstart send qcovs evalue bitscore'); 3) all blast hits with low confidence scores (alignment length < 200 nn; Identity < 90%; query coverage < 100%; Gaps > 0), OTUs with more than one taxonomic genus blast hit assigned, and/or classified as “uncultured, unidentified, unclassified or environmental”, were removed from the analyses; 4) lastly, in dubious cases (e.g. two different species hits to the same OTU) the blast parameters were re-evaluated (On this stage was performed a ranking of the blast parameters ; 1st Percentage Identity; 2nd E-value; 3rd Percentage of query coverage; 4th number of Gaps) and at the same time OTUs were manually scrutinized/selected via alignment (OTU vs. both references, using the Jalview tool^62^. In the latter case, the dubious OTU’s were classified based on the alignment quality against the references; 5) for the remaining OTUs the taxonomy classification was based on the top hit.

#### NIS detection

NIS detection was only based on the COI sequences. The COI OTUs, previously classified, were compared against the following reference databases: the Global Invasive Species Database (GISD: https://www.iucngisd.org/gisd/), the Invasive Species Compendium (CABI: www.cabi.org/isc), the Information system on aquatic non-indigenous and cryptogenic species (AquaNIS: www.corpi.ku.lt/databases/index.php/aquanis/) and the National Exotic Marine and Estuarine Species Information System (NEMESIS: //invasions.si.edu/nemesis/ ), all accessed on March 2020. All positive hits were validated using two approaches. First, COI sequences were processed in Translator X server^63^ using variable genetic codes and following NCBI tables. All sequences without translation and/or with stop codons were removed from the analyses. Second, all sequences with positive matches and encoding an interrupted protein were subject to manual curation, through alignment at the protein and nucleotide levels, against the reference.

### Statistical analyses

The Alpha-diversity indices (Observed OTUs, Chao1, Shannon, and Inverse-Simpson) for the four-gene markers were calculated using the Phyloseq package (version 1.5.3)^64^ and the sample rarefaction curves were calculated with the vegan package^65^ (version 2.5–5), both in RStudio, R statistical software (version 2.15.2)^66^. A one-way ANOVA analysis was used to compare the diversity indices values, plates biomass, and the abundance of fouling organisms for the treatments (presence vs. absence of paint). If the data did not follow a normal distribution (analyzed with the Shapiro–Wilk normality test), differences among treatments were analyzed using the non-parametric Kruskal–Wallis test, followed by a two-tailed Mann–Whitney-Wilcoxon test for pairwise comparisons. All statistical data analysis was performed using R statistical software^66^ (version 2.15.2). A multivariate version of Levene’s analysis of multivariate homogeneity of group dispersions (function ‘betadisper’ in R vegan package^65^) was used to test for homogeneity of variances of the groups (plates with paint vs plates without paint). A permutational multivariate analysis of variance (PERMANOVA)^67^ was subsequently used to test the taxonomic dissimilarity between the plates with anti-corrosion paint and the plates without anti-corrosion paint. For the PERMANOVA analysis, Bray–Curtis resemblance matrices were produced using the untransformed raw data, and p-values were calculated from 9,999 random permutations and dummy variables (0.0001) were used to account for zero. This test was performed using the ‘adonis’ function provided by the vegan package^65^ and ran in the R statistical software^66^.

## Results

### Morphological identification and monitoring of the biofouling community

**Figure 2:**
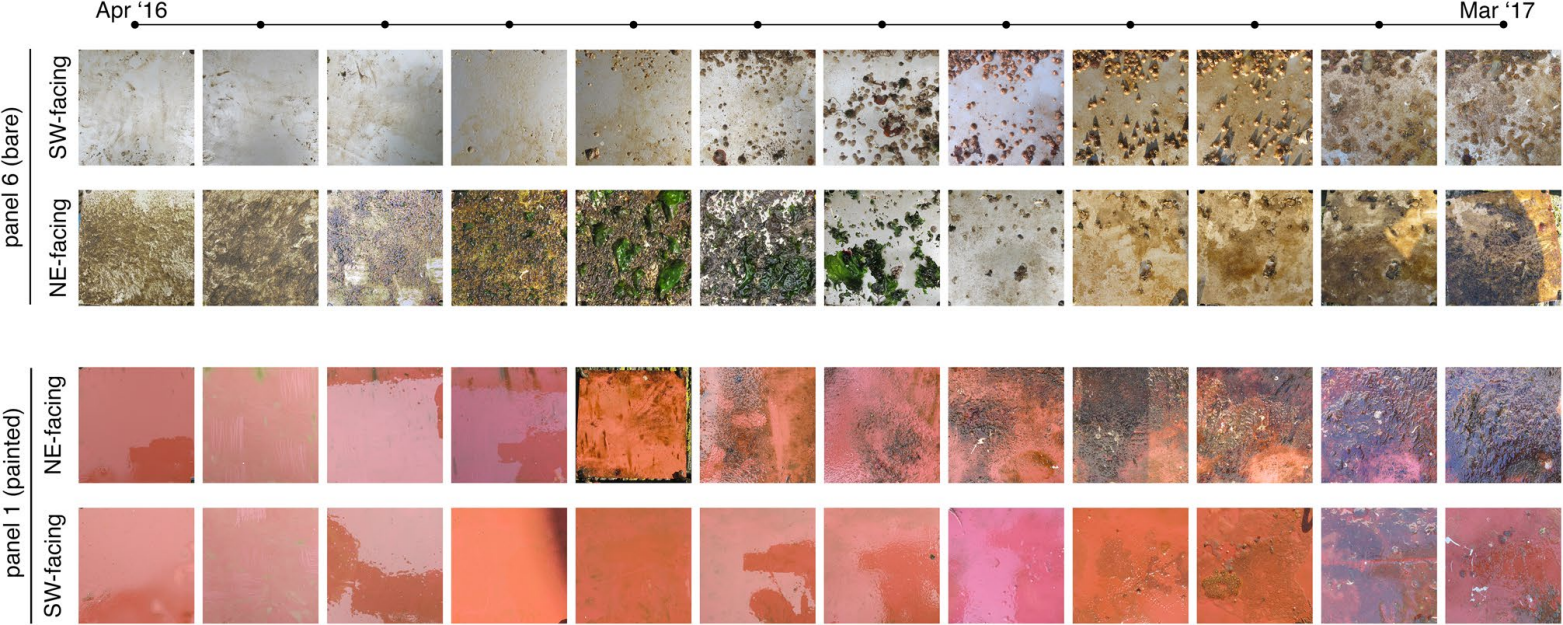
Temporal development of marine biofouling. Photographs of bare panel 6 (P6) and painted panel 1 (P1) facing Northeast (NE) and Southwest (SW).

Macro- and microscopic assessment of the submerged steel panels led to the identification of a vast number of both fouler and non-fouler organisms based on morphology. In total, about 28 different taxa, belonging to 12 phyla, 15 classes, 23 orders, 25 families, 27 genera and 11 described species were identified (Table S3). A clear difference between the Southwest and Northeast facing panels concerning colonization rate (Fig. 2) and main taxonomic composition was observed. The bare panels facing Southwest were firstly colonized by juveniles of the crustacean *Chthamalus* sp., followed by the bivalve *Mytilus* spp*.* (Fig. 2). From September to November 2016, the solitary ascidian *Ciona intestinalis*, the red ripple bryozoan *Watersipora subtorquata*, cnidarians, and polychaetes were the new colonizers. On the other hand, in December 2016, the painted panels facing southwest were colonized by bryozoans, particularly by the star ascidian *Botryllus schlosseri*. After one month, the bare panels facing the Northeast were completely covered by macroalgae, e.g. *Ectocarpus* sp. and *Ceramium cilliatum*, by a vast number of diatom species, as well as protozoa, red and brown algae, copepods, the barnacle *Chthamalus* sp. and *Mytilus* spp. mussel larvae. After the second month of deployment (May 2016), two hydrozoan genera, i.e. *Ectopleura* and *Obelia*, and the red alga *C. cilliatum* were also observed. During the summer period (June–August 2016) those plates were colonized by the green algae *Ulva* sp*.* and by the bell-shaped ciliate *Pseudovorticella* spp.. In the last three months (January to March 2017), macroalgae and crustacean larvae were observed again (Fig. 2; Table S3).

**Figure 3:**
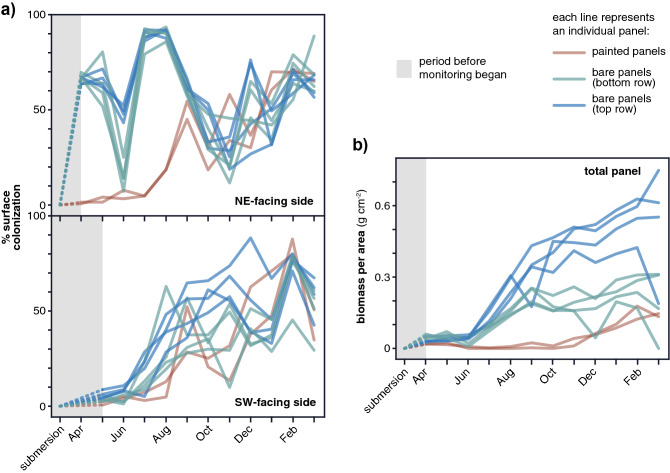
Monitoring of biofouling progression and biomass determination: (**a**) Surface colonization assessed by ImageJ on both panels facing sides. Note: Southwest facing side panel image analysis only started in May; (**b**) Biomass per area monitored during the 12 months. For both figures, each line represents an individual panel.

The percentage of surface colonization, assessed by computer-assisted image analysis, indicated significant differences between painted and bare panels, as well as between the panels facing Northeast or Southwest (Fig. 3a). As expected, the bare panels were the first to be colonized, and fouling covered a higher percentage of the surface when compared to the painted panels (Fig. 3a). Regarding the entire plates (both Northeast- and Southwest-facing sides), in the first month of sampling, there was an average coverage of 34.7% and 2.0% for bare and painted panels (P < 0.01), respectively (Fig. 3a). Around the sixth month of colonization, a plateau with no significant differences in coverage area between bare and painted plates was reached (Fig. 3a). Interestingly, in the last month of sampling, only slight differences between both types of plates were observed with painted plates showing a higher surface area covered (84.1%) in comparison with bare plates (72.5%) (Fig. 3a). Regarding the colonization per orientation, the bare panels facing Southwest, had an average of 65.1% of colonization in the first month while the Northeast bare panels showed significantly lower colonization of 4.2% (P < 0.01) (Fig. 3a). After the 12 months, similar fractions of the covered surface were observed: Southwest (73.4%) and Northeast (71.7%) (Fig. 3a). In terms of biomass, the painted panels had an average biomass colonization per area of 0.2 g/cm^2^, while the bare panels on the top and the bottom row of colonization showed about 0.6 and 0.3 g/cm^2^, respectively) (Fig. 3b). Thus, the presence of paint in the plates was associated with a significantly lower amount of biomass (p < 0.01).

### Metabarcoding analysis of community biodiversity

The four gene markers used in this metabarcoding approach (16S rRNA, 18S rRNA, 23S rRNA, and COI gene markers) were successfully amplified for almost all samples. The only exception was the COI gene, which did not amplify for one painted panel sample (February 2017; data not shown). The amount of DNA per sample ranged from 12.9 to 114 (ng/μL). The quality control steps of the bioinformatic approach showed some quality differences among markers. While the 16S rRNA and 18S rRNA quality filtering saw a relatively small fraction of reads removed, the 23S rRNA and COI genes datasets were drastically reduced, from 1,167,426 to 332,609 (23S rRNA) and from 1,464,221 to 250,245 (COI gene). The main factor for this reduction was the Q > 20 parameter (> 80% of total reads removed from both datasets). The average length of the merged reads (as counted using the fastx-info command of usearch) was of 310 bp for 16S rRNA, 300 bp for 18S rRNA, 368 bp for 23S rRNA and 313 bp for COI. As expected, the number of OTUs assigned were also highly uneven: 7,329 OTUs for the 16S rRNA, 40,092 OTUs for the 18S rRNA gene, 925 OTUs for the 23S rRNA gene, and 1,614 OTUs for the COI gene all based on 97% similarity (Table 1). To perform the taxonomy assignment, we have applied a homology-based approach. In this approach we have used the top 5 BLAST hits, though acknowledging their limitations in terms of accuracy and some level of uncertainty that is unaccounted in dubious cases. To guarantee the robustness of the results and mitigate the above mentioned points we have used solid and restrict parameters. As a result, the metabarcoding strategy allowed the identification of 665 different eukaryotic species in 23S rRNA and COI data (28 for the 23S rRNA gene and 637 for the COI gene) which used the same taxonomic assignment methodology. A high level of taxonomy assignment was obtained for three out of the four markers. For the 16S rRNA gene, all OTU sequences (100%) were successfully assigned against the Greengenes database, while for the 18S rRNA and COI genes, 91.5 and 90.0% of sequences, respectively, were classified using the SILVA, NCBI or BOLD databases. On the other hand, the taxonomy assignment of the 23S rRNA gene data was only possible for 33.2% of the sequences using the NCBI or BOLD database.

**Table 1:** Overview of the bioinformatics workflow data for the Illumina Miseq V3-V4 sequencing (300 bp paired-end read) of the 16S rRNA, 18S rRNA, 23S rRNA, and COI genes.

Target gene	16S rRNA	18S rRNA	23S rRNA	COI
Raw read pairs	3,035,203	2,960,440	1,590,577	1,891,088
Merged reads	2,401,893	2,420,421	1,167,426	1,464,221
Number of sequences after filtering (> 100 bp; Q > 20; chimeric)	2,093,546	2,169,662	332,609	250,245
Number of OTUs (97% similarity)	7,329	40,092	925	1,614
Unassigned OTUs	0	3,415	618	161
Bacterial OTUs	7,329	0	292	27
Bacterial genera	620	0	92	13
Bacterial species	–	–	1	13
Eukaryotic OTUs	0	36,667	215	1,426
Eukaryotic genera	0	282	174	482
Eukaryotic species	–	–	28	637

**Figure 4:**
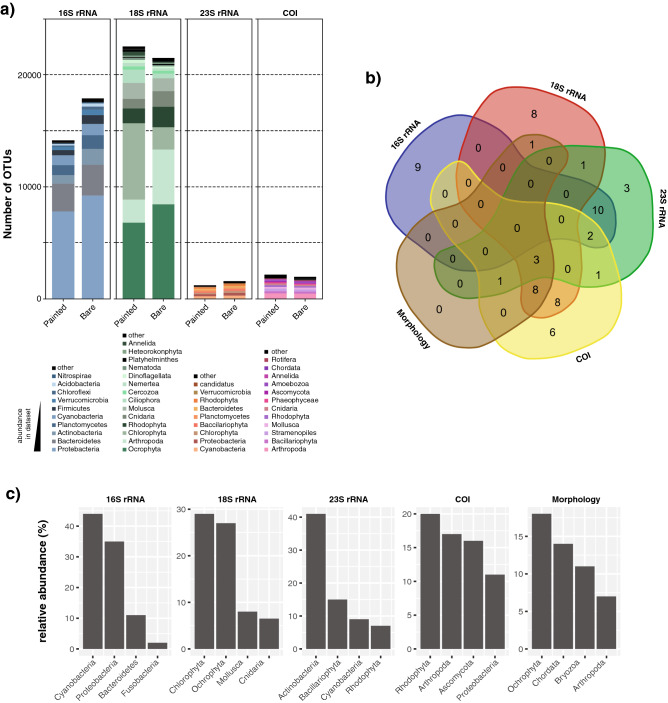
(**a**) Overview of fouling communities in the painted and bare panels considering the total OTUs obtained from 16S rRNA, 18S rRNA, 23S rRNA, and COI; (**b**) Venn diagram (bioinformatics.psb.ugent.be/webtools/Venn/) showing exclusive and shared phyla between the four gene markers and morphology; (**c**) Most abundant phyla, corresponding to the respective number of taxonomic assignments compared to the total taxonomic assignments in the different OTU tables or in the morphological identification table (see text for details).

The total number of OTUs for the bare and painted panels (Fig. 4a) among the same markers demonstrated that the total number of OTUs for the 18S rRNA and COI genes is higher in the painted panels, while for the 16S and 23S rRNA OTUs the total number of OTUs is higher in the bare panels.

One of the main advantages of the multi-marker approach concerns the detection of exclusive phyla per marker (i.e. phyla that are only observed in the dataset of a particular marker). In this study, a total of twenty-six phyla were exclusively detected using a single marker: nine phyla with the 16S rRNA gene; eight phyla for the 18S rRNA gene; three phyla for 23S rRNA gene and six phyla for the COI gene, but no exclusive phyla identified using the morphological identification (Fig. 4b, Table S4). Non-exclusive phyla were naturally also identified for each marker (16S rRNA (12), 18S rRNA (21), 23S rRNA (18) and COI (22), while 13 phyla were identified using microscopy (Fig. 4b). The most abundant phyla for each marker were: Cyanobacteria (44%) for 16S rRNA; Chlorophyta (29%) for 18S rRNA; Actinobacteria (41%) for 23S rRNA, and Rhodophyta (20%) for COI, and Ochrophyta (18%) was the most abundant in the morphological identification (Fig. 4c). Taking into account the global set of assigned bacterial OTUs, it is possible to verify that the 16S rRNA gene marker was the largest contributor with 620 genera, followed by the 23S rRNA data (92 genera and 1 species) and COI (13 genera and 13 species). On the other hand, the majority of eukaryotic OTUs were found in COI data (482 genera and 637 species), followed by the 18S rRNA data (282 genera) and the 23S rRNA data (174 genera and 28 species).

### Diversity analyses and temporal variation of the most abundant groups

Regarding alpha-diversity, no significant differences were found for any of the tested indices (Observed OTUs, Chao1, Shannon, and Inverse-Simpson) for each of the four markers when comparing the diversity between the plates with and without the employed anti-corrosion paint. The dispersion between groups using the betadisper test was not significant for any of the gene marker dataset, meaning that group dispersions are homogenous (p > 0.05) for all the gene marker datasets. The subsequent PERMANOVA analysis demonstrated a significant dissimilarity between the taxa growing in surfaces with and without anti-corrosion paint, both for the 16S rRNA (p = 0.014) (Table 2) and the 23S rRNA amplicon data (p = 0.005) (Table 2). On the other hand, there was no significant effect of the anti-corrosion paint on the relative taxonomic abundance obtained for the 18S rRNA and COI data (p > 0.05) (Table 2).

**Table 2:** PERMANOVA test results based on Bray–Curtis (BC) similarity measure for square-root transformed relative abundances of the OTUs for the four-gene markers dataset.

	*df*	Sums of sqs	Mean Sqs	F. Model	R^2^	Pr(> F)
**16S rRNA**						
Treatment	1	0.6096	0.60955	1.8883	0.07905	0.014*
Residuals	22	7.1018	0.32281	–	0.92095	–
Totals	23	7.7113	–	1.00000	–	–
**18S rRNA**						
Treatment	1	0.5406	0.54058	1.4035	0.05997	0.083
Residuals	22	8.4734	0.38515	–	0.94003	–
Totals	23	9.0139	–	9.0139	–	–
**23S rRNA**						
Treatment	1	0.8453	0.84525	2.432	0.09954	0.005**
Residuals	22	7.6462	0.34755	–	0.90046	–
Totals	23	8.4914	–	1.00000	–	–
**COI**						
Treatment	1	0.5215	0.52150	1.1328	0.05118	0.212
Residuals	21	9.6675	0.46036	–	0.46036	–
Totals	22	10.1890	–	1.00000	–	–

Signif. codes: ‘**’ 0.01 ‘*’ 0.05 ‘.’

**Figure 5:**
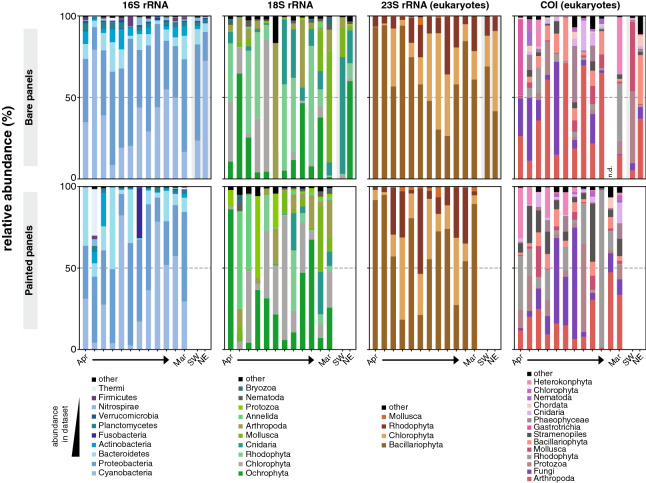
Relative abundance analyses of 16S rRNA, 18S rRNA, 23S rRNA, and COI gene marker, at the phylum level of painted and bare panels.

Regarding prokaryotes, the 16S rRNA gene dataset retrieved, as expected, a higher number of OTUs for all the phyla when compared to all other markers (p < 0.01) except for the Firmicutes and Verrumicrobia phyla, for which no significant differences were found when compared to the 23S rRNA gene data (Fig. 4a). The relative abundance of 16S rRNA gene-derived OTUs demonstrated that the most dominant phyla for both painted and bare panels were Cyanobacteria (42.6%), Proteobacteria (38.9%), Bacteroidetes (9.2%) and Actinobacteria (2.9%) (Fig. 4b). The 16S rRNA gene data revealed that the anaerobic phylum Fusobacteria was abundant on the painted panel sampled in October (Fig. 5) (30.9%), while in the other months its abundance was much lower (always lower than 2%) (p < 0.01) (Fig. 5). The dominant phyla retrieved from the 16S rRNA gene dataset at the Northeast-facing side were Cyanobacteria (72.5%) and Bactoroidetes (5.1%) while at the Southwest-facing side the dominant colonizer was Proteobacteria (59.1%). The Bacteroidetes and Chloroflexi phyla were more abundant in plates with anti-corrosion paint (p < 0.05), while Firmicutes were significantly more abundant in the plates without anti-corrosion paint (p < 0.05). No other significant differences at the phyla level for the 16S rRNA gene were found when comparing painted and bare panels. As for temporal differences, a significant increase of Cyanobacteria with colonization time was observed for the 16S rRNA gene data (from 34.8% in the first month until 43.4% in the tenth month of sampling) (p < 0.05), but these differences were not observed for the same phylum in the 23S rRNA dataset (Fig. 5).

In terms of eukaryotic OTUs (Fig. 4a), no major differences for the abundance of taxa were found between the 18S rRNA, 23S rRNA, and COI gene markers, except for the phylum Cnidaria which is significantly more abundant in the 18S rRNA gene dataset (8.1%) compared with the COI dataset (2.3%, p < 0.05), the phylum Stramenopiles which is higher for the COI (5.0%) compared to 18S rRNA dataset(0.05%, p < 0.01). Likewise, Chlorophyta (green algae) had a higher abundance in the 23S rRNA gene (7.8%) and 18S rRNA gene data (29.8%) compared with the COI dataset (1.1%, all differences p < 0.05) (Fig. 4c). As for the effect of the paint, we could observe in the 18S rRNA gene data that the abundance of the phylum Mollusca is significantly higher in the bare panels compared to the panels with paint (p < 0.05) (17.2% compared to 9.8%), but these differences were not observed for the COI dataset (Fig. 4b). Regarding the temporal succession combining both plates with and without paint no major differences were found at the phylum level for all the gene markers (Fig. 5). However, when the plates with and without paint are analyzed separately, significant differences were observed. Namely, the 18S rRNA gene marker revealed that the phylum Ochrophyta was the dominant colonizer of the painted panels since the second month of colonization (87.1%), while Chlorophyta (36.3%) Rhodophyta (33.1%), were the more abundant phyla in the bare panels (Fig. 5). In the same dataset, the abundance of the freshwater green algae Charophyta (p < 0.05) was significantly higher in the plates without anti-corrosion paint (4.2%) than in the plates with the paint (2.3%), while the Protozoa group was significantly higher in plates with paint (6.2%) compared to the bare panels (3.1%) (p < 0.05). As for the effect of the orientation of the plates, the phyla recovered from the Northeast-facing side with the 18S rRNA gene marker were mainly Chlorophyta (28.9%), and Ochrophyta (27.6%) while from the Southwest-facing side the three more abundant colonizer groups were Arthropoda (12.1%), Mollusca (7.6%), and Cnidaria (5.3%) (Fig. 5). The data obtained using the COI gene marker retrieved mainly phyla Ochrophyta (21.2%), Rhodophyta (13.4%) and Protozoa (34.1%) in the painted panels, while the most abundant phylum in the bare panels was Arthropoda (20.1%) (Fig. 5). The main taxa retrieved from the Southwest-facing side were Mollusca (32.1%), while on the Northeast-facing side the most prevalent were Arthropoda (30.3%) and Annelida (17.6%) (Fig. 5). Regarding the algae, there was a higher abundance of Chlorophyta in the early months of colonization for the bare panels only in the COI gene dataset (11.3%), while the for Rhodophyta had higher colonization in the last three months for both 18S rRNA and COI genes (23.3% and 15.6%) (p < 0.05) (Fig. 5). Similar to the prokaryotic data, the 23S rRNA gene data did not reveal any major temporal differences or any effect of the paint on the abundance of the eukaryotic phyla.

### NIS present in Port of Leixões

Potential NIS were only detected among the COI-derived OTUs. During the taxonomic classification of COI gene-derived OTUs, two databases (NT-NCBI and BOLD) were used. Despite our effort to classify the OTUs sequences without classification in NT-NCBI database, against BOLD database, no new classifications were found. In summary, 27 species, representing 1.7% of the total retrieved COI gene OTUs, were found to be potential NIS (Table S5). Three of these are on the list of global invader species recognized by the Invasive Species Specialist Group of the International Union of Conservation of Nature (IUCN)^68^, namely the red algae *Polysiphonia brodiei*, the incrusting bryozoan *Watersipora subtorquata* and the barnacle *Austrominius modestus*. Two NIS were also morphologically identified, i.e., the hydroid *Ectopleura crocea* and the bryozoan *Watersipora subtorquata*. All the NIS found are tertiary colonizers and macrofoulers, except for five species, namely the Bristly crab *Pilumnus hirtellus,* the copepod *Oithona similis*, the mudshrimp *Monocorophium sextonae*, and the annelids *Ctenodrilus serratus* and *Chaetogaster diaphanus*. The Australian tubeworm *Ficopomatus enigmaticus*^69^ (Table S3) and the solitary ascidian *Styela clava*, a native species from the Northwest Pacific, and the only previously reported NIS for this port^70,71^. Both these species were exclusively identified using the morphological approach. All the COI-derived OTUs (nucleotide sequences) that resulted in NIS identification, as well as the blast-hits results used for the taxonomic classification of NIS, are provided in Supplementary Table S6 and Supplementary File S1.

## Discussion

In this work, we used both DNA-dependent and independent techniques to monitor the biofouling dynamics in a Portuguese port for one year. The metabarcoding approach employed four different gene markers, which minimizes taxonomic bias^8,72^ and is less likely to under-detect small organisms^73^. By the same token, the taxonomic classification pipelines continue to be computationally intensive and highly dependent on huge computational resources. Due to the computational limitations we have applied a homology-based approach to perform the taxonomic classification and used conservative parameters to mitigate their limitations in terms of accuracy. Overall, our multi-marker metabarcoding data confirms that this is a powerful tool to assess biofouling community biodiversity, enhancing the resolution of taxa by providing richer taxonomic information about this Portuguese port. Nevertheless, the detection of nonindigenous species was only achieved with one of the gene markers (COI) employed in the study. Furthermore, and despite the clear advantages of metabarcoding approaches for the monitoring of micro- and macro-foulers, there are problems inherent to such approaches, ranging from primer bias^74^ to differences in resolution among markers, to the lack of reference databases and universal bioinformatics pipelines to deal with next-generation sequencing (NGS)-generated datasets^75^. Nevertheless, our findings demonstrate that all employed markers added important data to the biofouling survey by detecting unique taxonomic groups. A high level of marine diversity detection was achieved by the metabarcoding approach: we retrieved 7,329 OTUs for 16S rRNA, 40,092 OTUs for 18S rRNA, 925 OTUs for 23S rRNA and 1,614 OTUs for COI. Overall, the choice of data filtering, clustering algorithms, and specific parameters can have a significant impact on the obtained OTUs for the different genes employed in the metabarcoding approach. Results based on length variable regions, such as the 18S rRNA, are strongly influenced by the data processing workflow such as the clustering methods which can result in the overestimation of OTU numbers^76^. As for the detected species, COI retrieved more species (637 species) than the 23S rRNA marker (28 species) and COI was the only gene contributing to the detection of NIS in the Port of Leixões. The deficiency of taxonomic coverage in reference databases is one of the major obstacles to large-scale application of metabarcoding in biodiversity research and biofouling monitoring^17,29^. In this study, for each of the gene markers we used one, or a combination of two databases (Greengenes, SILVA, BOLD, and NT-NCBI) taking in consideration the respective gene and the target taxa to be amplified (Table S2).

The metabarcoding approach revealed to be much more efficient in retrieving taxa at low taxonomic levels than the morphologic approach – we identified a total of 1,704 genera and a possible total of 611 species (excluding bacteria) compared to the 28 genera and 11 species which were morphologically identified. It should be stated that taxonomy assignment at the lowest levels, particularly at a species level has some degree of uncertainty due to the restricted specificity/resolution of markers and incompleteness of currently available reference sequence databases. The morphological approach employed here did not provide any new information to assess biofouling community biodiversity, at the phylum level, in the sampled port. Nevertheless, important biofouling organisms, such as the macroalgae *Ulva* sp.*,* the solitary and colonial ascidians (*Styela Clava*, *Ciona intestinalis, Dendrodoa* sp. and *Botryllus* sp.) and the polychaeta *Ficopomatus enigmatus* were only visually detected. The absence of these prevalent groups in the metabarcoding data could probably be explained by the natural complexity of marine biofouling matrices, which may not have allowed complete homogeneity of the samples before DNA extraction^29^. Another explanation could be the randomly chosen panel grids used in our sampling. Hence, our findings indicate that morphological identification is still a valuable strategy to be implemented in monitoring programs to identify fouling and NIS species. Almost all rarefaction curves for the alpha diversity reached the saturation plateau (Fig. S1), suggesting that this study sampling effort resulted in the appropriate sequencing.

Regarding the effect of the anti-corrosion paint, there is the absence of significant differences for any of the alpha-diversity indices (Observed OTUs, Chao1, Shannon and Inverse-Simpson) for all the markers. On the other hand, the PERMANOVA analysis results demonstrated that for the 16S rRNA and 23S rRNA gene markers, there was a strong dissimilarity between the communities growing in the presence and the absence of paint, but this was not observed for the other markers. Some previous studies employing commercial anti-fouling coatings reported a lower diversity of the fouling communities as a result of the presence of those commercial products in the surfaces^77–80^, with a more significant effect in the prokaryotic communities than in the eukaryotic communities^22,30^ Proteobacteria, Cyanobacteria, and Bacteroidetes are known to be the dominant bacterial phyla colonizing different surfaces at diverse sampling locations, particularly in the early stages of colonization^81,82^. In our study, we found a significantly higher presence of the phylum Bacteroidetes in the plates with anti-corrosion paint when compared to the plates without paint. Bacteroidetes are efficient surface colonizers of steel surfaces in the marine environment and are known to be associated with biological corrosion^2,82,83^. Moreover, Firmicutes are other well-known colonizers of immersed artificial surfaces^84^, and here we report a significantly higher abundance of this phylum in the plates without anti-corrosion paint. Usually, studies characterizing prokaryotic communities colonizing artificial surfaces involve sampling attached communities growing for weeks or a few months^1^, while our work involved a longer sampling period which could explain why for the bare panels such a high abundance of sequences was classified as cyanobacteria/plastids in the Greengenes database. The particular dominance of photoautotrophic organisms in the panels facing Northeast is also in line with the effect of sunlight in their colonization. At the same time, the colonization of these groups was limited in the plates with anti-corrosion paint and delayed for approximately six months, which is in line with previous studies^78,79^.

Regarding the eukaryotic communities, Rhodophyta, Chlorophyta, Ochrophyta, Cnidaria, Arthropoda, and Mollusca were the most abundant taxa along the year, which is consistent with previous macrofouling studies^22^. The data from the 18S rRNA and 23S rRNA gene markers suggests a community stabilization that initiated in the fourth month of the deployment of the panels, whereby the main taxonomic groups demonstrate little variation in their abundance until the last sampling month. In terms of seasonal patterns, and as revealed by 18S rRNA gene data, green algae (Chlorophyta) are dominant in Spring and Summer in the bare panels when compared to other algae (Rhodophyta or Ochrophyta) in agreement with previous studies^85^. Among the marine fouling communities, red algae (Rhodophyta) are believed to settle after the hard-foulers and filamentous algae^3^, and in our study, the settlement of these organisms initiated after a few months of the immersion of the plates.

Regarding hard-fouler colonization, a higher presence of barnacles in the Southwest-facing plates (with no direct exposure to the sunlight) was observed when compared to the Northeast facing plates (with direct exposure to the sunlight). On the other hand, there was a more abundant and earlier colonization of algae in the surfaces facing Northeast (See Figs. 1 and 2). This may be explained by the fact that photosynthesis-able fouling biomass is usually limited to well-irradiated areas whereas barnacle larvae are typically found to be negatively phototactic^3^. The COI gene marker data revealed more extensive colonization by the phototrophic taxonomic group Bacillariophyta in the Northeast panels, with the Southwest panels showing the dominance of Arthropoda. Visual inspection indicated that barnacles (*Chthamalus* sp. and *Amphibalanus* sp.), and mussels (*Mytilus* spp.) were dominant all year in all plates. Other hard-fouler organisms like bryozoans are more reported in temperate climates during the warmer seasons of Spring or in Summer, and our findings are in line with this, particularly for the 18S rRNA gene dataset^86^. The results from the 18S rRNA and COI gene markers reveal stronger colonization of Cnidaria in the Summer, with increased abundance for the Spring season, similar to previous studies^87^. No significant presence of sponges was detected in our results, a group that is often reported in biofouling assemblages^87–89^. In general, no consistent seasonal patterns of abundance were identified among most of the analyzed taxa, suggesting stabilization of communities after the early months of immersion^90–92^. Nevertheless, our data clearly shows that, as expected, bare panels harbour higher biomass than the painted panels, which means that the employed commercial anti-corrosion paint has a significant impact in the colonization of the communities in employed steel plates. A significant effect of the paint in the early colonization of the communities was also observed. This difference decreased during the sampling period and converged with the levels of bare panels around the tenth month of colonization. Regarding the rate of colonization, panels facing northwest showed a total coverage of around 100% in the fifth month of sampling, with a decline of surface coverage in the subsequent months. On the other hand, panels facing Northeast were never fully colonized, reaching close to 90% coverage in the ninth month of sampling. Previous studies from fouling communities sampled from temperate areas suggest a rapid increase in colonization reaching 75–100% after one year, whereas at higher latitudes this colonization process is much slower^93^. Regarding the colonization of biomass per area, there were significantly higher biomass colonization values for the bare panels, with higher access to light, also likely due to the importance of phototrophic organisms in the structuring of the fouling community.

Finally, a list of 29 NIS present in the Port of Leixões was for the first time compiled in this work (27 NIS from metabarcoding and 2 NIS from morphological approach), after comparing our outputs with the ICES, ISSG, GISD, CABI, AquaNIS and NEMESIS databases. Currently, baseline studies of biofouling monitoring programs are being globally conducted and implemented at ports^19^ as the colonization analysis and identification of biofouling species will facilitate the development of antifouling strategies^94^. However, future improvements are needed for the implementation of these approaches as part of the routine port species monitorization. In agreement with the Port Biological Baseline Survey (PBBS) we selected the sampling location in a high priority area in Northern Portugal. For correct monitoring of fouling communities, PBBS suggests taking in consideration the seasonality and life cycle patterns of those species and conduct sampling in certain seasons of the year, when species are mature and can be identified by morphology. However, metabarcoding approaches have allowed the detection of conspicuous organisms as well of unseen organisms^18,95^ which was also the case in our study. Furthermore, the year-long approach to the monitoring of the fouling species revealed to be important for future monitoring studies as some taxonomic shifts were detected. As expected, our results underline that morpho-taxonomy identification is particularly well suited for the detection of conspicuous organisms, such as macrofauna or macroalgae which can be readily identified but are occasionally not detected by the metabarcoding approach. Lastly, and taking in consideration our results, we suggest that the development of a regional reference database of non-indigenous species would enhance the metabarcoding approach accuracy for future monitoring studies.

## Conclusions

Biofouling monitoring of ports gathers essential information for the planning and the maintenance of marine structures and allows for early NIS identification. Here, we report the biofouling diversity and their succession in the Port of Leixões for one year using a combined morphological and multi-marker metabarcoding approach. These approaches proved to be complementary since a higher number of retrieved OTUs were obtained with the metabarcoding approach while some of the NIS were only visually detected. Key to our analysis was the use of four different gene markers and different reference databases. This allowed for a high reported diversity and has the additional benefit of enabling cross-verification of species detections, such as for rare or non-indigenous species of interest. This study also includes the first published NIS list of the Port of Leixões which can be a starting point for future biofouling monitoring programs. Hence, we conclude that a combination of morphological and metabarcoding multi-marker methodologies is an advantageous strategy for biofouling monitoring programs and the detection of potential NIS in future studies.

## Supplementary information

Supplementary information

## Data Availability

All the data processed and discussed during this study are public and free available in the main manuscript and in supplementary files. Additionally, all the sequencing datasets were deposited in the European Nucleotide Archive (ENA) under the accession number PRJEB33602.
