# Supplementary material for: Monitoring of biofouling communities in a Portuguese port using a combined morphological and metabarcoding approach
Source: Sci Rep. 2020 Aug 10;10:13461. doi: 10.1038/s41598-020-70307-4 (PMC7417558; doi:10.1038/s41598-020-70307-4)
Supplement: Supplementary file 1 — Supplementary information [file 41598_2020_70307_MOESM1_ESM.pdf]

Supplementary information

Monitoring of biofouling communities in a Portuguese port using a combined morphological and metabarcoding approach

Joana Azevedo[1,2a], Jorge Antunes[1,2a], André M. Machado[1], Vitor Vasconcelos[1,2], Pedro Leão[1,*] and Elsa Froufe[1,*]

[1] Interdisciplinary Centre of Marine and Environmental Research, CIIMAR/CIMAR, Matosinhos, Portugal

[2] Faculty of Sciences, University of Porto, Portugal

[a] authors contributed equally to this work

*corresponding authors

This document includes:

<u>Supplementary Tables 1-6</u>

Supplementary Table S1. Summary of the samples surveyed per month, respective biomass and DNA concentration.

Supplementary Table S2. Summary of the primers used to amplify the main target fouling taxa present on the monitoring structure.

Supplementary Table S3. Morphological identification of the main taxa observed in the stainless-steel plates deployed at Leixões port from April 2016 until March 2017. Values 0 and 1 indicate absence and presence, respectively.

Supplementary Table S4. List of detected phyla with the different approaches.

Supplementary Table S5. List of NIS found in the COI NGS datasets.

Supplementary Table S6. Blast-hit results used for the taxonomic assignment of NIS. All blast hit results come from the search of the COI-Derived OTUs in NT-NCBI database.

<u>Supplementary Figures 1</u>

Supplementary Figure S1. Sampling–month based rarefaction curves of Chao 1 for both painted and bare panels and for each of the four gene markers. Dashed lines indicate 95% confidence interval.

<u>Supplementary File S1</u>

Supplementary File S1. Nucleotide sequences of OTUs found in the COI-derived dataset and with correspondence in at least one of the four NIS databases Inspected.

Supplementary Table S1. Summary of the samples surveyed per month, respective biomass and DNA concentration.

Sampling per Month	Grids	Subsamples	Sequenced Sample	Biomass of Sequenced Sample (g)	DNA of Sequenced Sample (ng/μL)
April 2016	6 painted grids	3 painted subsamples	1 painted sample	0.1602	23 ± 3.1
	12 bare grids	3 bare subsamples	1 bare sample	0.2539	26.9 ±8.0
May 2016	6 painted grids	3 painted subsamples	1 painted sample	0.0867	114 ± 16.0
	12 bare grids	3 bare subsamples	1 bare sample	0.1663	62.7 ± 6.4
Jun 2016	6 painted grids	3 painted subsamples	1 painted sample	0.0162	87.5 ± 1.90
	12 bare grids	3 bare subsamples	1 bare sample	0.0492	47.2 ± 1.02
Jul 2016	6 painted grids	3 painted subsamples	1 painted sample	0.0166	42.8 ± 3.48
	12 bare grids	3 bare subsamples	1 bare sample	0.2509	50.6 ± 3.96
Aug 2016	6 painted grids	3 painted subsamples	1 painted sample	0.0236	67.9 ± 2.66
	12 bare grids	3 bare subsamples	1 bare sample	0.1559	56.9 ± 1.02
Sep 2016	6 painted grids	3 painted subsamples	1 painted sample	0.0162	69.5 ± 4.14
	12 bare grids	3 bare subsamples	1 bare sample	0.3144	81.7 ± 0.47
Oct 2016	6 painted grids	3 painted subsamples	1 painted sample	0.0344	57.1 ± 1.16
	12 bare grids	3 bare subsamples	1 bare sample	0.0858	40.1 ± 0.95
Nov 2016	6 painted grids	3 painted subsamples	1 painted sample	0.1398	18.0 ± 0.79
	12 bare grids	3 bare subsamples	1 bare sample	0.2635	60.6 ± 1.17
Dez 2016	6 painted grids	3 painted subsamples	1 painted sample	0.4162	27.4 ± 1.35
	12 bare grids	3 bare subsamples	1 bare sample	0.8132	22.3 ± 0.13
Jan 2017	6 painted grids	3 painted subsamples	1 painted sample	0.2183	68.7 ± 1.09
	12 bare grids	3 bare subsamples	1 bare sample	0.3370	23.7 ± 3.45
Feb 2017	6 painted grids	3 painted subsamples	1 painted sample	0.1195	24.5 ± 4.3
	12 bare grids	3 bare subsamples	1 bare sample	0.2756	34.2 ± 0.16
Mar 2017	6 painted grids	3 painted subsamples	1 painted sample	0.2121	21.9 ± 0.11
	12 bare grids	3 bare subsamples	1 bare sample	0.6403	12.9 ± 2.17
Mar 2017 Northeast	hole plate	1 bare subsample	1 bare sample	0.5298	14.8 ± 0.07
Mar 2017 Southwest	Whole plate	1 bare subsample	1 bares ample	0.6330	22.3 ± 0.07

Supplementary Table S2. Summary of the primers used to amplify the main target fouling taxa present on the monitoring structure.

Gene marker	Primer set	Primer Sequence (5'-3')	Fragment size (bp)	Target taxa
16S rRNA	341F [49]	CCTACGGGNGGCWGCAG	464	Prokaryotes
	785R [49]	GACTACHVGGGTATCTAATCC		
18S rRNA	TAReuk454FWD1 [50]	CCAGCASCYGCGGTAATTCC	270	Eukaryotes
	TAReukREV3 [50]	ACTTTCGTTCTTGATYRATGA		
COI	mlCOIintF [51]	TAAACTTCAGGGTGACCAAARAAYCA	313	Metazoans
	dgHCO2198 [52]	GGWACWGGWTGAACWGTWTAYCCYCC		
23S rRNA	p23SrVF [53]	GGACAGAAAGACCCTATGAA	377	Eukaryotic algae and cyanobacteria
	p23SrVR [53]	TCAGCCTGTTATCCCTAGAG		

Supplementary Table S3. Morphological identification of the main taxa observed in the stainless-steel plates deployed at Leixões port from April 2016 until March 2017. Values 0 and 1 indicate absence and presence, respectively.

Observed Genus/Species	*Sampling months from April 2016 to March 2017*											
	April	May	June	July	Aug.	Sept.	Out.	Nov.	Dec.	Jan.	Feb.	March
Actinia sp.	0	0	0	0	0	0	1	1	1	1	1	1
Amoeba sp.	0	0	0	0	0	0	0	1	0	0	0	0
Amphibalanus sp.	1	1	1	1	1	1	1	1	1	1	1	1
Asterionellopsis sp.	1	1	1	1	1	1	1	1	1	1	1	1
Bacillaria sp.	1	1	1	1	1	1	1	1	1	1	1	1
Botryllus schlosseri	0	0	0	0	0	0	0	0	1	1	1	1

Bugula sp.	0	1	1	1	0	0	0	0	0	0	0	0
Callochiton sp.	0	0	0	0	0	0	0	0	0	0	0	1
Ceranium cilliatum	0	1	1	1	0	0	0	0	0	0	0	1
Chthamalus sp.	1	1	1	1	1	1	1	1	1	1	1	1
Ciona intestinalis	0	0	0	0	0	1	1	1	1	1	1	1
Dendrodoa grossularia	0	0	0	0	0	0	0	0	0	0	1	1
Didemnum vexillum	0	0	0	0	0	0	0	0	0	0	1	1
Ectocarpus sp.	1	1	1	1	0	0	0	0	0	1	1	1
Ectopleura sp.	0	1	0	0	0	0	0	0	1	1	1	1

Ficopomatus enigmatus	0	0	1	1	1	1	1	1	1	1	1	1
Gelidium sp.	0	0	0	0	0	0	1	1	1	1	1	1
Licmophora sp.	1	1	1	1	1	1	1	1	1	1	1	1
Melosira artica	1	1	1	1	1	1	1	1	1	1	1	1
Melosira nummuloides	1	1	1	1	1	1	1	1	1	1	1	1
Mytilus sp.	1	1	1	1	1	1	1	1	1	1	1	1
Obelia sp.	0	1	1	1	0	0	0	0	0	0	0	0
Paracentrotus lividus	1	1	1	1	1	1	1	1	1	1	1	1
Pseudvorticella sp.	1	0	1	1	1	0	0	0	0	0	1	1

Sabellastarte sp.	0	0	0	0	0	0	1	1	1	1	1	1
Striatella sp.	1	1	1	1	1	1	1	1	1	1	1	1
Ulva lactuca	0	0	0	1	1	1	1	0	0	0	0	0
Watersipora subatra	0	0	0	0	0	1	1	1	1	1	1	1

Supplementary Table S4. List of detected phyla with the different approaches.

Phyla	16S rRNA	18S rRNA	23S rRNA	COI	Morphology
Acidobacteria	Acidobacteria	-	Acidobacteria	-	-
Actinobacteria	Actinobacteria	-	Actinobacteria	-	-
Alveolata	-	-	Alveolata	-	-
Amoebozoa	-	Amoebozoa	-	Amoebozoa	Amoebozoa
Annelida	-	Annelida	-	Annelida	Annelida
Apicomplexa	-	Apicomplexa	-	-	-
Apusozoa	-	Apusozoa	-	-	-
Arthropoda	-	Arthropoda	-	Arthropoda	Arthropoda
Ascomycota	-	-	-	Ascomycota	-
Bacillariophyta	-	-	Bacillariophyta	Bacillariophyta	Bacillariophyta
Bacteroidetes	Bacteroidetes	-	Bacteroidetes	Bacteroidetes	-
Basidiomycota	-	-	-	Basidiomycota	-
Brachiopoda	-	-	-	Brachiopoda	-
Bryozoa	-	Bryozoa	-	Bryozoa	Bryozoa
Candidatus	-	-	Candidatus	-	-
Cercozoa	-	Cercozoa	-	-	-
Charophyta	-	Charophyta	-	-	-
Chlamydiae	Chlamydiae	-	-	-	-
Chlorobi	Chlorobi	-	-	-	-
Chloroflexi	Chloroflexi	-	Chloroflexi	-	-
Chlorophyta	-	Chlorophyta	Chlorophyta	Chlorophyta	Chlorophyta
Chordata	-	Chordata	-	Chordata	Chordata
Ciliophora	-	Ciliophora	-	-	Ciliophora
Cnidaria	-	Cnidaria	-	Cnidaria	Cnidaria
Crenarchaeota	Crenarchaeota	-	-	-	-
Cryptophyta	-	Cryptophyta	Cryptophyta	-	-
Cyanobacteria	Cyanobacteria	-	Cyanobacteria	-	-
Dinoflagellata	-	Dinoflagellata			
Echinodermata	-	Echinodermata	-	Echinodermata	Echinodermata
Euglenozoa	-	-	Euglenozoa	-	-
Euryarchaeota	Euryarchaeota	-	-	-	-
Excavata	-	Excavata	-	-	-
Firmicutes	Firmicutes	-	Firmicutes	-	-
Fusobacteria	Fusobacteria	-	-	-	-
Gastrotricha	-	Gastrotricha	-	Gastrotricha	-
Gemmatimonadetes	Gemmatimonadetes	-	-	-	-
Haptophyta	-	Haptophyta	-	Haptophyta	-
Lentisphaerae	Lentisphaerae	-	Lentisphaerae	-	-
Mollusca	-	Mollusca	Mollusca	Mollusca	Mollusca
Nematoda	-	Nematoda	-	Nematoda	-
Nemertea	-	Nemertea	-	Nemertea	-
Nitrospirae	Nitrospirae	-	Nitrospirae	-	-
Ochrophyta	-	Ochrophyta	-	-	Ochrophyta
Phaeophyceae	-	-	-	Phaeophyceae	-
Placozoa	-	-	-	Placozoa	-
Planctomycetes	Planctomycetes	-	Planctomycetes	Platyhelminthes	-
Platyhelminthes	-	Platyhelminthes	-	-	-

Porifera	-	Porifera	-	Porifera	-
Proteobacteria	Proteobacteria	-	Proteobacteria	Proteobacteria	-
Protozoa	-	Protozoa	-	-	-
Rhodophyta	-	Rhodophyta	Rhodophyta	Rhodophyta	Rhodophyta
Rhombozoa	-	-	-	Rhombozoa	-
Rotifera	-	Rotifera	-	Rotifera	-
Spirochaetes	Spirochaetes	-	-	-	-
Stramenopiles	-	Stramenopiles	-	Stramenopiles	-
Streptophyta	-	-	Streptophyta	Streptophyta	-
Synergistetes	Synergistetes	-	-	-	-
Tardigrada	-	-	-	Tardigrada	-
Tenericutes	Tenericutes	-	Tenericutes	-	-
Thermi	Thermi	-	-	-	-
Verrucomicrobia	Verrucomicrobia	-	Verrucomicrobia	-	-

Supplementary Table S5. List of NIS found in the COI NGS datasets.

Taxa	Common Name	Phylum	Native Range	Reference
Aiptasia pulchella	Glass anemone	Cnidaria	NW Pacific	http://www.corpi.ku.lt/databases/index.php/aquanis/species/view/id/523
Amathia gracilis	Moss animal	Bryozoa	NE Atlantic	http://www.corpi.ku.lt/databases/index.php/aquanis/species/view/id/544
Amphibalanus improvisus	Bay barnacle	Arthropoda	NW Atlantic	http://www.corpi.ku.lt/databases/index.php/aquanis/species/view/id/537
Austrominius modestus	Balanus	Arthropoda	Pacific	http://www.corpi.ku.lt/databases/index.php/aquanis/species/view/id/480
Bougainvillia muscus	Umbrella semi-globular	Cnidaria	North Atlantic Ocean including Mediterranean Sea; Arctic Ocean; E and W South Atlantic and Indo-Pacific Oceans	http://invasions.si.edu/nemesis/browseDB/SpeciesSummary.jsp?TSN=-29
Callyspongia siphonella	Tube sponge	Porifera	Red Sea	http://www.marinespecies.org/porifera/porifera.php?p=taxdetails&id=166199
Ceramium secundatum	-	Rhodophyta	Northern Atlantic Ocean	http://invasions.si.edu/nemesis/browseDB/GroupSummary.jsp?GRP=Algae https://www.marlin.ac.uk/species/detail/1476
Chaetogaster diaphanus	-	Annelida	New Zealand Exclusive Economic Zone; North Atlantic Ocean; Poland; Spanish Exclusive Economic Zone	http://invasions.si.edu/nemesis/calnemo/searchTaxa.jsp?taxon=Chaetogaster+diaphanus http://www.catalogueoflife.org/annual-checklist/2019/details/species/id/baca38822a630349a645ad5fe1ed7d37
Clytia hemisphaerica	Jellyfish	Cnidaria	North America	https://www.cabi.org/isc/datasheet/113386
Ctenodrilus serratus	-	Annelida	Mediterranean Sea	http://www.corpi.ku.lt/databases/index.php/aquanis/species/view/id/1747
Dasya baillouviana	-	Rhodophyta	Mediterranean NE Atlantic	http://www.corpi.ku.lt/databases/index.php/aquanis/species/view/id/746
Ectopleura crocea	Pink-mouth hydroid	Cnidaria	NW Atlantic	https://animaldiversity.org/accounts/Ectopleura_crocea/

Halichondria panicea	Breadcrumb sponge	Porifera	North Atlantic; Mediterranean Sea	http://invasions.si.edu/nemesis/browseDB/searchTaxa.jsp?taxon=Halichondria+panicea
Haliclystus tenuis	Stalked jellyfish	Cnidaria	Antarctic Atlantic; Southwest Atlantic and Southeast Pacific	http://www.corpi.ku.lt/databases/index.php/aquanis/species/view/id/1715
Halothrix lumbricalis	-	Ochrophyta	Distribution Long Island Sound to Gulf of Maine,Scotian Shelf to Gulf of St. Lawrence, and to the northeastern Newfoundland Shelf	http://www.corpi.ku.lt/databases/index.php/aquanis/species/view/id/485 http://www.marinespecies.org/aphia.php?p=taxdetails&id=144944#notes
Hymeniacidon perlevis	Crumb-of-bread sponge	Porifera	Northern Atlantic Ocean: Western Europe	http://www.corpi.ku.lt/databases/index.php/aquanis/species/view/id/1725 http://www.marinespecies.org/aphia.php?p=taxdetails&id=132663#distributions
Monocorophium sextonae	Mudshrimp	Arthropoda	New Zealand or NE Atlantic depending of information source	http://www.corpi.ku.lt/databases/index.php/aquanis/species/view/id/566 https://www.nobanis.org/globalassets/speciesinfo/m/monocorophium-sextonae/monocorophium-sextonae3.pdf
Myrionema strangulans	-	Chordariaceae	Long Island Sound to Gulf of St. Lawrence, Strait of Belle Isle	http://invasions.si.edu/nemesis/browseDB/searchTaxa.jsp?taxon=Myrionema+strangulans http://www.marinespecies.org/aphia.php?p=taxdetails&id=144985#notes
Oithona similis	Copepod	Arthropoda	Distribution: Arctic to Cape Cod	http://www.corpi.ku.lt/databases/index.php/aquanis/species/view/id/981/ http://www.marinespecies.org/aphia.php?p=taxdetails&id=106656#notes

Perforatus perforatus	Acorn barnacle	Arthropoda	Distribution: Mediterranean and extending southward to the coasts of Africa and northwards up the Spanish and French coasts to the south-west coasts of Great Britain	http://www.corpi.ku.lt/databases/index.php/aquanis/species/view/id/1642/ https://www.marlin.ac.uk/species/detail/1603
Pilumnus hirtellus	Bristly crab	Arthropoda	Distribution: Southern Norway southwards to Cape Verde Islands, Mediterranean and Black Sea	http://www.corpi.ku.lt/databases/index.php/aquanis/species/view/id/1511 https://www.marlin.ac.uk/species/detail/1627
Polysiphonia brodiei *	-	Rhodophyta	Distribution: UK and Scandinavia	https://www.cabi.org/isc/datasheet/107751
Polysiphonia denudata	-	Rhodophyta	Western Indian Ocean and Western Central Atlantic	http://invasions.si.edu/nemesis/calnemo/GroupSummary.jsp?GRP=Algae / https://www.sealifebase.ca/summary/Polysiphonia-denudata.html#
Polysiphonia morrowii	-	Rhodophyta	North Pacific	http://www.corpi.ku.lt/databases/index.php/aquanis/species/view/id/919
Porphyra umbilicalis	Black butter	Rhodophyta	Mediterranean Sea	http://www.corpi.ku.lt/databases/index.php/aquanis/species/view/id/1431
Ruditapes philippinarum	Japanese cockle	Mollusca	China and Japan	http://www.corpi.ku.lt/databases/index.php/aquanis/species/view/id/500
*Watersipora subtorquata**	Moss animal	Bryozoa	Gulf of Mexico	http://www.corpi.ku.lt/databases/index.php/aquanis/species/view/id/689

*Species identified in both metabarcoding and morphologically approach.

Supplementary Table S6. Blast-hit results used for the taxonomic assignment of NIS. All blast hit results come from the search of the COI-Derived OTUs in NT-NCBI database.

OTU id	NT-NCBI Accession number	Species	Alignment Lenght	Identity (%)	GAPS	Coverage (%)	E-value	Bitscore	Taxon Id	Description in NT-NCBI
Uniq199;size=360;	gi|537743898	*Aiptasia pulchella*	312	95.513	0	100	1.85E-137	499	12924	Aiptasia pulchella complete mitochondrial genome, isolate US1
Uniq199;size=360;	gi|537743883	*Aiptasia pulchella*	312	95.513	0	100	1.85E-137	499	12924	Aiptasia pulchella complete mitochondrial genome, isolate NOR1
Uniq211;size=328;	gi|537743898	*Aiptasia pulchella*	312	95.513	0	100	1.85E-137	499	12924	Aiptasia pulchella complete mitochondrial genome, isolate US1
Uniq211;size=328;	gi|537743883	*Aiptasia pulchella*	312	95.513	0	100	1.85E-137	499	12924	Aiptasia pulchella complete mitochondrial genome, isolate NOR1
Uniq13879;size=6;	gi|1383527676	*Amathia gracilis*	308	93.506	0	98	3.13E-125	459	560980	Amathia gracilis voucher BMBM-0907 cytochrome oxidase subunit 1 (COI) gene, partial cds; mitochondrial
Uniq480;size=130;	gi|1035662449	*Amphibalanus improvisus*	312	99.679	0	100	3.86E-159	571	1220549	Amphibalanus improvisus voucher USNM:IZ:1287238 cytochrome oxidase subunit 1 (COI) gene, partial cds; mitochondrial
Uniq480;size=130;	gi|936253556	*Amphibalanus improvisus*	312	99.679	0	100	3.86E-159	571	1220549	Amphibalanus improvisus voucher MT03225 cytochrome oxidase subunit 1 (COI) gene, partial cds; mitochondrial
Uniq480;size=130;	gi|936253512	*Amphibalanus improvisus*	312	99.679	0	100	3.86E-159	571	1220549	Amphibalanus improvisus voucher MT03221 cytochrome oxidase subunit 1 (COI) gene, partial cds; mitochondrial
Uniq480;size=130;	gi|936253088	*Amphibalanus improvisus*	312	99.679	0	100	3.86E-159	571	1220549	Amphibalanus improvisus voucher MT03223 cytochrome oxidase subunit 1 (COI) gene, partial cds; mitochondrial

**Uniq480;size=130;**	gi|786321723	*Amphibalanus improvisus*	312	99.679	0	100	3.86E-159	571	1220549	Amphibalanus improvisus isolate Bsex-353 cytochrome oxidase subunit I (COI) gene, partial cds; mitochondrial
**Uniq512;size=122;**	gi|1035662449	*Amphibalanus improvisus*	312	99.679	0	100	3.86E-159	571	1220549	Amphibalanus improvisus voucher USNM:IZ:1287238 cytochrome oxidase subunit 1 (COI) gene, partial cds; mitochondrial
**Uniq512;size=122;**	gi|936253556	*Amphibalanus improvisus*	312	99.679	0	100	3.86E-159	571	1220549	Amphibalanus improvisus voucher MT03225 cytochrome oxidase subunit 1 (COI) gene, partial cds; mitochondrial
**Uniq512;size=122;**	gi|936253512	*Amphibalanus improvisus*	312	99.679	0	100	3.86E-159	571	1220549	Amphibalanus improvisus voucher MT03221 cytochrome oxidase subunit 1 (COI) gene, partial cds; mitochondrial
**Uniq512;size=122;**	gi|936253088	*Amphibalanus improvisus*	312	99.679	0	100	3.86E-159	571	1220549	Amphibalanus improvisus voucher MT03223 cytochrome oxidase subunit 1 (COI) gene, partial cds; mitochondrial
**Uniq512;size=122;**	gi|786321723	*Amphibalanus improvisus*	312	99.679	0	100	3.86E-159	571	1220549	Amphibalanus improvisus isolate Bsex-353 cytochrome oxidase subunit I (COI) gene, partial cds; mitochondrial
**Uniq246;size=265;**	gi|1320903723	*Austrominius modestus*	312	99.679	0	100	3.86E-159	571	1732093	Austrominius modestus isolate 010316-06C cytochrome c oxidase subunit I (COI) gene, partial cds; mitochondrial
**Uniq246;size=265;**	gi|1304472505	*Austrominius modestus*	312	99.679	0	100	3.86E-159	571	1732093	Austrominius modestus isolate 010316_06MM cytochrome c oxidase subunit I (COI) gene, partial cds; mitochondrial
**Uniq246;size=265;**	gi|936252898	*Austrominius modestus*	312	99.679	0	100	3.86E-159	571	1732093	Austrominius modestus voucher MT03161 cytochrome oxidase subunit 1 (COI) gene, partial cds; mitochondrial

**Uniq246;size=265;**	gi|1320903727	*Austrominius modestus*	312	99.359	0	100	1.80E-157	566	1732093	Austrominius modestus isolate 010316-06B cytochrome c oxidase subunit I (COI) gene, partial cds; mitochondrial
**Uniq246;size=265;**	gi|936253892	*Austrominius modestus*	312	99.359	0	100	1.80E-157	566	1732093	Austrominius modestus voucher MT03158 cytochrome oxidase subunit 1 (COI) gene, partial cds; mitochondrial
**Uniq279;size=227;**	gi|1320903723	*Austrominius modestus*	311	99.678	0	99	1.39E-158	569	1732093	Austrominius modestus isolate 010316-06C cytochrome c oxidase subunit I (COI) gene, partial cds; mitochondrial
**Uniq279;size=227;**	gi|1304472505	*Austrominius modestus*	311	99.678	0	99	1.39E-158	569	1732093	Austrominius modestus isolate 010316_06MM cytochrome c oxidase subunit I (COI) gene, partial cds; mitochondrial
**Uniq279;size=227;**	gi|936252898	*Austrominius modestus*	311	99.678	0	99	1.39E-158	569	1732093	Austrominius modestus voucher MT03161 cytochrome oxidase subunit 1 (COI) gene, partial cds; mitochondrial
**Uniq279;size=227;**	gi|1320903727	*Austrominius modestus*	311	99.357	0	99	6.46E-157	564	1732093	Austrominius modestus isolate 010316-06B cytochrome c oxidase subunit I (COI) gene, partial cds; mitochondrial
**Uniq279;size=227;**	gi|936253892	*Austrominius modestus*	311	99.357	0	99	6.46E-157	564	1732093	Austrominius modestus voucher MT03158 cytochrome oxidase subunit 1 (COI) gene, partial cds; mitochondrial
**Uniq7;size=27913;**	gi|985560044	*Bougainvillia muscus*	312	100	0	100	8.29E-161	577	308567	Bougainvillia muscus isolate 345 cytochrome oxidase subunit I (COI) gene, partial cds; mitochondrial
**Uniq8;size=27711;**	gi|985560044	*Bougainvillia muscus*	312	100	0	100	8.29E-161	577	308567	Bougainvillia muscus isolate 345 cytochrome oxidase subunit I (COI) gene, partial cds; mitochondrial
**Uniq10583;size=8;**	gi|664803921	*Callyspongia siphonella*	297	97.306	0	95	3.84E-139	505	469330	Callyspongia siphonella cytochrome oxidase subunit I (COI) gene, partial cds; mitochondrial

**Uniq13537;size=6;**	gi|1122285008	*Callyspongia siphonella*	294	98.639	0	94	3.94E-144	521	469330	Callyspongia siphonella isolate V2K1 cytochrome c oxidase subunit I (COI) gene, partial cds; mitochondrial
**Uniq6503;size=14;**	gi|664803921	*Callyspongia siphonella*	296	97.297	0	94	1.43E-138	503	469330	Callyspongia siphonella cytochrome oxidase subunit I (COI) gene, partial cds; mitochondrial
**Uniq7265;size=12;**	gi|1122285008	*Callyspongia siphonella*	295	98.644	0	94	1.11E-144	523	469330	Callyspongia siphonella isolate V2K1 cytochrome c oxidase subunit I (COI) gene, partial cds; mitochondrial
**Uniq1420;size=54;**	gi|961526476	*Ceramium secundatum*	312	94.551	0	100	1.86E-132	483	193556	Ceramium secundatum voucher GWS018041 cytochrome oxidase subunit 1 (COI) gene, partial cds; mitochondrial
**Uniq2314;size=36;**	gi|961526476	*Ceramium secundatum*	312	94.551	0	100	1.86E-132	483	193556	Ceramium secundatum voucher GWS018041 cytochrome oxidase subunit 1 (COI) gene, partial cds; mitochondrial
**Uniq4695;size=19;**	gi|378532182	*Chaetogaster diaphanus*	312	97.756	0	100	3.91E-149	538	212246	Chaetogaster diaphanus isolate CE439 cytochrome oxidase subunit I (COI) gene, partial cds; mitochondrial
**Uniq4695;size=19;**	gi|793346027	*Chaetogaster diaphanus*	312	97.115	0	100	8.47E-146	527	212246	Chaetogaster diaphanus mitochondrial partial COI gene for cytochrome oxidase subunit 1, isolate N1_251
**Uniq6528;size=14;**	gi|378532182	*Chaetogaster diaphanus*	312	97.756	0	100	3.91E-149	538	212246	Chaetogaster diaphanus isolate CE439 cytochrome oxidase subunit I (COI) gene, partial cds; mitochondrial
**Uniq6528;size=14;**	gi|793346027	*Chaetogaster diaphanus*	312	97.115	0	100	8.47E-146	527	212246	Chaetogaster diaphanus mitochondrial partial COI gene for cytochrome oxidase subunit 1, isolate N1_251
**Uniq86;size=966;**	gi|1069323480	*Clytia hemisphaerica*	312	100	0	100	8.29E-161	577	252671	Clytia hemisphaerica voucher MZUSP:2795 cytochrome oxidase

										subunit I (COI) gene, partial cds; mitochondrial
**Uniq86;size=966;**	gi|1069323456	*Clytia hemisphaerica*	312	99.679	0	100	3.86E-159	571	252671	Clytia hemisphaerica voucher MZUSP:2790 cytochrome oxidase subunit I (COI) gene, partial cds; mitochondrial
**Uniq86;size=966;**	gi|1069323472	*Clytia hemisphaerica*	312	99.359	0	100	1.80E-157	566	252671	Clytia hemisphaerica voucher MZUSP:2794 cytochrome oxidase subunit I (COI) gene, partial cds; mitochondrial
**Uniq86;size=966;**	gi|1069323460	*Clytia hemisphaerica*	312	99.359	0	100	1.80E-157	566	252671	Clytia hemisphaerica voucher MZUSP:2792 cytochrome oxidase subunit I (COI) gene, partial cds; mitochondrial
**Uniq86;size=966;**	gi|1069323468	*Clytia hemisphaerica*	312	99.038	0	100	8.35E-156	560	252671	Clytia hemisphaerica voucher MZUSP:2793 cytochrome oxidase subunit I (COI) gene, partial cds; mitochondrial
**Uniq87;size=949;**	gi|1069323480	*Clytia hemisphaerica*	312	100	0	100	8.29E-161	577	252671	Clytia hemisphaerica voucher MZUSP:2795 cytochrome oxidase subunit I (COI) gene, partial cds; mitochondrial
**Uniq87;size=949;**	gi|1069323456	*Clytia hemisphaerica*	312	99.679	0	100	3.86E-159	571	252671	Clytia hemisphaerica voucher MZUSP:2790 cytochrome oxidase subunit I (COI) gene, partial cds; mitochondrial
**Uniq87;size=949;**	gi|1069323472	*Clytia hemisphaerica*	312	99.359	0	100	1.80E-157	566	252671	Clytia hemisphaerica voucher MZUSP:2794 cytochrome oxidase subunit I (COI) gene, partial cds; mitochondrial
**Uniq87;size=949;**	gi|1069323460	*Clytia hemisphaerica*	312	99.359	0	100	1.80E-157	566	252671	Clytia hemisphaerica voucher MZUSP:2792 cytochrome oxidase subunit I (COI) gene, partial cds; mitochondrial
**Uniq87;size=949;**	gi|1069323468	*Clytia hemisphaerica*	312	99.038	0	100	8.35E-156	560	252671	Clytia hemisphaerica voucher MZUSP:2793 cytochrome oxidase

										subunit I (COI) gene, partial cds; mitochondrial
**Uniq2123;size=39;**	gi|874520343	*Ctenodrilus serratus*	312	98.718	0	100	3.89E-154	555	40316	Ctenodrilus serratus cytochrome oxidase subunit I gene, partial cds; mitochondrial
**Uniq161;size=472;**	gi|1198411024	*Dasya baillouviana*	303	94.389	0	97	1.87E-127	466	35166	Dasya baillouviana voucher TRP/CWS 12-166-1 [BDA1776] cytochrome oxidase subunit 1 (COI) gene, partial cds; mitochondrial
**Uniq172;size=448;**	gi|1198411024	*Dasya baillouviana*	302	94.371	0	96	6.74E-127	464	35166	Dasya baillouviana voucher TRP/CWS 12-166-1 [BDA1776] cytochrome oxidase subunit 1 (COI) gene, partial cds; mitochondrial
**Uniq681;size=96;**	gi|1198411024	*Dasya baillouviana*	303	92.739	0	97	4.08E-119	438	35166	Dasya baillouviana voucher TRP/CWS 12-166-1 [BDA1776] cytochrome oxidase subunit 1 (COI) gene, partial cds; mitochondrial
**Uniq537;size=116;**	gi|1198411024	*Dasya baillouviana*	302	92.715	0	96	1.47E-118	436	35166	Dasya baillouviana voucher TRP/CWS 12-166-1 [BDA1776] cytochrome oxidase subunit 1 (COI) gene, partial cds; mitochondrial
**Uniq4519;size=20;**	gi|527468477	*Ectopleura crocea*	312	100	0	100	8.29E-161	577	576744	Ectopleura crocea isolate Acro01PR cytochrome c oxidase subunit I (COI) gene, partial cds; mitochondrial
**Uniq4519;size=20;**	gi|527468473	*Ectopleura crocea*	312	99.679	0	100	3.86E-159	571	576744	Ectopleura crocea isolate Acro03JU cytochrome c oxidase subunit I (COI) gene, partial cds; mitochondrial
**Uniq4519;size=20;**	gi|527468467	*Ectopleura crocea*	312	99.679	0	100	3.86E-159	571	576744	Ectopleura crocea isolate Acro01RJ cytochrome c oxidase subunit I (COI) gene, partial cds; mitochondrial

Uniq4519;size=20;	gi|527468475	*Ectopleura crocea*	312	99.359	0	100	1.80E-157	566	576744	Ectopleura crocea isolate Acro05JU cytochrome c oxidase subunit I (COI) gene, partial cds; mitochondrial
Uniq4519;size=20;	gi|527468471	*Ectopleura crocea*	312	99.359	0	100	1.80E-157	566	576744	Ectopleura crocea isolate Acro04MA cytochrome c oxidase subunit I (COI) gene, partial cds; mitochondrial
Uniq7168;size=12;	gi|527468477	*Ectopleura crocea*	312	100	0	100	8.38E-161	577	576744	Ectopleura crocea isolate Acro01PR cytochrome c oxidase subunit I (COI) gene, partial cds; mitochondrial
Uniq7168;size=12;	gi|527468473	*Ectopleura crocea*	312	99.679	0	100	3.90E-159	571	576744	Ectopleura crocea isolate Acro03JU cytochrome c oxidase subunit I (COI) gene, partial cds; mitochondrial
Uniq7168;size=12;	gi|527468467	*Ectopleura crocea*	312	99.679	0	100	3.90E-159	571	576744	Ectopleura crocea isolate Acro01RJ cytochrome c oxidase subunit I (COI) gene, partial cds; mitochondrial
Uniq7168;size=12;	gi|527468475	*Ectopleura crocea*	312	99.359	0	100	1.81E-157	566	576744	Ectopleura crocea isolate Acro05JU cytochrome c oxidase subunit I (COI) gene, partial cds; mitochondrial
Uniq7168;size=12;	gi|527468471	*Ectopleura crocea*	312	99.359	0	100	1.81E-157	566	576744	Ectopleura crocea isolate Acro04MA cytochrome c oxidase subunit I (COI) gene, partial cds; mitochondrial
Uniq135;size=692;	gi|1321068866	*Halichondria panicea*	312	100	0	100	8.29E-161	577	6063	Halichondria panicea voucher SB16A cytochrome oxidase subunit I (COI) gene, partial cds; mitochondrial
Uniq135;size=692;	gi|512764451	*Halichondria panicea*	312	99.359	0	100	1.80E-157	566	6063	Halichondria panicea cytochrome oxidase subunit I (COI) gene, partial cds; mitochondrial
Uniq139;size=676;	gi|1321068866	*Halichondria panicea*	312	100	0	100	8.29E-161	577	6063	Halichondria panicea voucher SB16A cytochrome oxidase

										subunit I (COI) gene, partial cds; mitochondrial
**Uniq139;size=676;**	gi|512764451	*Halichondria panicea*	312	99.359	0	100	1.80E-157	566	6063	Halichondria panicea cytochrome oxidase subunit I (COI) gene, partial cds; mitochondrial
**Uniq141;size=668;**	gi|1025756842	*Haliclystus tenuis*	309	98.706	0	99	1.81E-152	549	756010	Haliclystus tenuis isolate 06JAP101-5 cytochrome oxidase subunit 1 (cox1) gene, partial cds; mitochondrial
**Uniq2061;size=40;**	gi|1025756842	*Haliclystus tenuis*	311	98.714	0	99	5.03E-153	551	756010	Haliclystus tenuis isolate 06JAP101-5 cytochrome oxidase subunit 1 (cox1) gene, partial cds; mitochondrial
**Uniq79;size=1063;**	gi|1025756842	*Haliclystus tenuis*	309	97.735	0	99	1.82E-147	532	756010	Haliclystus tenuis isolate 06JAP101-5 cytochrome oxidase subunit 1 (cox1) gene, partial cds; mitochondrial
**Uniq84;size=1010;**	gi|1025756842	*Haliclystus tenuis*	309	97.735	0	99	1.82E-147	532	756010	Haliclystus tenuis isolate 06JAP101-5 cytochrome oxidase subunit 1 (cox1) gene, partial cds; mitochondrial
**Uniq188;size=384;**	gi|781789938	*Halothrix lumbricalis*	310	100	0	99	1.07E-159	573	133519	Halothrix lumbricalis mitochondrial partial COI gene for cytochrome oxidase subunit 1, strain NAP12-s#3-30A
**Uniq188;size=384;**	gi|781786344	*Halothrix lumbricalis*	310	100	0	99	1.07E-159	573	133519	Halothrix lumbricalis mitochondrial partial COI gene for cytochrome oxidase subunit 1, strain GR11-54
**Uniq191;size=375;**	gi|781789938	*Halothrix lumbricalis*	309	100	0	99	3.86E-159	571	133519	Halothrix lumbricalis mitochondrial partial COI gene for cytochrome oxidase subunit 1, strain NAP12-s#3-30A
**Uniq191;size=375;**	gi|781786344	*Halothrix lumbricalis*	309	100	0	99	3.86E-159	571	133519	Halothrix lumbricalis mitochondrial partial COI gene for cytochrome oxidase subunit 1, strain GR11-54
**Uniq3833;size=23;**	gi|1321068858	*Hymeniacidon perlevis*	312	100	0	100	8.29E-161	577	177573	Hymeniacidon perlevis voucher Ag19A cytochrome oxidase

										subunit I (COI) gene, partial cds; mitochondrial
**Uniq3833;size=23;**	gi|1321068852	*Hymeniacidon perlevis*	312	100	0	100	8.29E-161	577	177573	Hymeniacidon perlevis voucher Ag19D cytochrome oxidase subunit I (COI) gene, partial cds; mitochondrial
**Uniq3833;size=23;**	gi|1321068848	*Hymeniacidon perlevis*	312	100	0	100	8.29E-161	577	177573	Hymeniacidon perlevis voucher Al17B cytochrome oxidase subunit I (COI) gene, partial cds; mitochondrial
**Uniq3833;size=23;**	gi|1321068846	*Hymeniacidon perlevis*	312	100	0	100	8.29E-161	577	177573	Hymeniacidon perlevis voucher Al17D cytochrome oxidase subunit I (COI) gene, partial cds; mitochondrial
**Uniq3833;size=23;**	gi|1321068840	*Hymeniacidon perlevis*	312	100	0	100	8.29E-161	577	177573	Hymeniacidon perlevis voucher An13C cytochrome oxidase subunit I (COI) gene, partial cds; mitochondrial
**Uniq6993;size=13;**	gi|1321068858	*Hymeniacidon perlevis*	312	100	0	100	8.38E-161	577	177573	Hymeniacidon perlevis voucher Ag19A cytochrome oxidase subunit I (COI) gene, partial cds; mitochondrial
**Uniq6993;size=13;**	gi|1321068854	*Hymeniacidon perlevis*	312	100	0	100	8.38E-161	577	177573	Hymeniacidon perlevis voucher Ag19C cytochrome oxidase subunit I (COI) gene, partial cds; mitochondrial
**Uniq6993;size=13;**	gi|1321068852	*Hymeniacidon perlevis*	312	100	0	100	8.38E-161	577	177573	Hymeniacidon perlevis voucher Ag19D cytochrome oxidase subunit I (COI) gene, partial cds; mitochondrial
**Uniq6993;size=13;**	gi|1321068848	*Hymeniacidon perlevis*	312	100	0	100	8.38E-161	577	177573	Hymeniacidon perlevis voucher Al17B cytochrome oxidase subunit I (COI) gene, partial cds; mitochondrial
**Uniq6993;size=13;**	gi|1321068846	*Hymeniacidon perlevis*	312	100	0	100	8.38E-161	577	177573	Hymeniacidon perlevis voucher Al17D cytochrome oxidase subunit

										I (COI) gene, partial cds; mitochondrial
**Uniq6230;size=14;**	gi|936254012	*Monocorophium sextonae*	308	100	0	98	1.39E-158	569	1582902	Monocorophium sextonae voucher MT01124 cytochrome oxidase subunit 1 (COI) gene, partial cds; mitochondrial
**Uniq6230;size=14;**	gi|936253422	*Monocorophium sextonae*	308	100	0	98	1.39E-158	569	1582902	Monocorophium sextonae voucher MT01123 cytochrome oxidase subunit 1 (COI) gene, partial cds; mitochondrial
**Uniq6230;size=14;**	gi|936252958	*Monocorophium sextonae*	308	100	0	98	1.39E-158	569	1582902	Monocorophium sextonae voucher MT01126 cytochrome oxidase subunit 1 (COI) gene, partial cds; mitochondrial
**Uniq6230;size=14;**	gi|936252870	*Monocorophium sextonae*	308	100	0	98	1.39E-158	569	1582902	Monocorophium sextonae voucher MT01127 cytochrome oxidase subunit 1 (COI) gene, partial cds; mitochondrial
**Uniq6328;size=14;**	gi|936254012	*Monocorophium sextonae*	309	100	0	99	3.86E-159	571	1582902	Monocorophium sextonae voucher MT01124 cytochrome oxidase subunit 1 (COI) gene, partial cds; mitochondrial
**Uniq6328;size=14;**	gi|936253422	*Monocorophium sextonae*	309	100	0	99	3.86E-159	571	1582902	Monocorophium sextonae voucher MT01123 cytochrome oxidase subunit 1 (COI) gene, partial cds; mitochondrial
**Uniq6328;size=14;**	gi|936252958	*Monocorophium sextonae*	309	100	0	99	3.86E-159	571	1582902	Monocorophium sextonae voucher MT01126 cytochrome oxidase subunit 1 (COI) gene, partial cds; mitochondrial
**Uniq6328;size=14;**	gi|936252870	*Monocorophium sextonae*	309	100	0	99	3.86E-159	571	1582902	Monocorophium sextonae voucher MT01127 cytochrome oxidase subunit 1 (COI) gene, partial cds; mitochondrial
**Uniq11718;size=7;**	gi|781789166	*Myrionema strangulans*	309	100	0	99	3.86E-159	571	86898	Myrionema strangulans mitochondrial partial COI gene for

										cytochrome oxidase subunit 1, strain BLZ12-18
Uniq11718;size=7;	gi|781788974	*Myrionema strangulans*	309	100	0	99	3.86E-159	571	86898	Myrionema strangulans mitochondrial partial COI gene for cytochrome oxidase subunit 1, strain BLZ11-233
Uniq11718;size=7;	gi|781788907	*Myrionema strangulans*	309	100	0	99	3.86E-159	571	86898	Myrionema strangulans mitochondrial partial COI gene for cytochrome oxidase subunit 1, strain BLZ11-219
Uniq11718;size=7;	gi|781788839	*Myrionema strangulans*	309	100	0	99	3.86E-159	571	86898	Myrionema strangulans mitochondrial partial COI gene for cytochrome oxidase subunit 1, strain BLZ11-204
Uniq11718;size=7;	gi|781788606	*Myrionema strangulans*	309	100	0	99	3.86E-159	571	86898	Myrionema strangulans mitochondrial partial COI gene for cytochrome oxidase subunit 1, strain BLZ11-171
Uniq5262;size=17;	gi|781789166	*Myrionema strangulans*	310	100	0	99	1.07E-159	573	86898	Myrionema strangulans mitochondrial partial COI gene for cytochrome oxidase subunit 1, strain BLZ12-18
Uniq5262;size=17;	gi|781788974	*Myrionema strangulans*	310	100	0	99	1.07E-159	573	86898	Myrionema strangulans mitochondrial partial COI gene for cytochrome oxidase subunit 1, strain BLZ11-233
Uniq5262;size=17;	gi|781788907	*Myrionema strangulans*	310	100	0	99	1.07E-159	573	86898	Myrionema strangulans mitochondrial partial COI gene for cytochrome oxidase subunit 1, strain BLZ11-219
Uniq5262;size=17;	gi|781788839	*Myrionema strangulans*	310	100	0	99	1.07E-159	573	86898	Myrionema strangulans mitochondrial partial COI gene for cytochrome oxidase subunit 1, strain BLZ11-204
Uniq5262;size=17;	gi|781788606	*Myrionema strangulans*	310	100	0	99	1.07E-159	573	86898	Myrionema strangulans mitochondrial partial COI gene for

										cytochrome oxidase subunit 1, strain BLZ11-171
Uniq5051;size=18;	gi|1024390348	*Oithona similis*	231	99.134	0	74	1.91E-112	416	324873	Oithona similis voucher OsiF1176_NA cytochrome c oxidase subunit I (COI) gene, partial cds; mitochondrial
Uniq5051;size=18;	gi|1024390346	*Oithona similis*	231	99.134	0	74	1.91E-112	416	324873	Oithona similis voucher 704Osi16_NA COI gene, partial sequence; mitochondrial
Uniq5051;size=18;	gi|1024390344	*Oithona similis*	231	99.134	0	74	1.91E-112	416	324873	Oithona similis voucher 484Osi23_NA cytochrome c oxidase subunit I (COI) gene, partial cds; mitochondrial
Uniq5051;size=18;	gi|1024390342	*Oithona similis*	231	99.134	0	74	1.91E-112	416	324873	Oithona similis voucher OsiF1183_NA cytochrome c oxidase subunit I (COI) gene, partial cds; mitochondrial
Uniq5051;size=18;	gi|1024390340	*Oithona similis*	231	99.134	0	74	1.91E-112	416	324873	Oithona similis voucher OsiF1182_NA COI gene, partial sequence; mitochondrial
Uniq106;size=801;	gi|949478689	*Perforatus perforatus*	276	100	0	88	8.53E-141	510	1562161	Perforatus perforatus cytochrome oxidase subunit 1 (COI) gene, partial cds; mitochondrial
Uniq106;size=801;	gi|1122285432	*Perforatus perforatus*	312	99.038	0	100	8.35E-156	560	1562161	Perforatus perforatus isolate F3S4 cytochrome c oxidase subunit I (COI) gene, partial cds; mitochondrial
Uniq106;size=801;	gi|1020994734	*Perforatus perforatus*	312	99.038	0	100	8.35E-156	560	1562161	Perforatus perforatus isolate 153472A cytochrome oxidase subunit 1 (COI) gene, partial cds; mitochondrial
Uniq127;size=719;	gi|949478689	*Perforatus perforatus*	275	100	0	88	3.07E-140	508	1562161	Perforatus perforatus cytochrome oxidase subunit 1 (COI) gene, partial cds; mitochondrial
Uniq127;size=719;	gi|1122285432	*Perforatus perforatus*	312	99.038	0	100	8.35E-156	560	1562161	Perforatus perforatus isolate F3S4 cytochrome c oxidase subunit I

										(COI) gene, partial cds; mitochondrial
**Uniq127;size=719;**	gi|1020994734	*Perforatus perforatus*	312	99.038	0	100	8.35E-156	560	1562161	Perforatus perforatus isolate 153472A cytochrome oxidase subunit 1 (COI) gene, partial cds; mitochondrial
**Uniq16100;size=5;**	gi|1122285440	*Pilumnus hirtellus*	312	100	0	100	8.29E-161	577	483407	Pilumnus hirtellus isolate K11 cytochrome c oxidase subunit I (COI) gene, partial cds; mitochondrial
**Uniq16100;size=5;**	gi|936254418	*Pilumnus hirtellus*	312	100	0	100	8.29E-161	577	483407	Pilumnus hirtellus voucher MT04581 cytochrome oxidase subunit 1 (COI) gene, partial cds; mitochondrial
**Uniq16100;size=5;**	gi|936254044	*Pilumnus hirtellus*	312	100	0	100	8.29E-161	577	483407	Pilumnus hirtellus voucher MT03176 cytochrome oxidase subunit 1 (COI) gene, partial cds; mitochondrial
**Uniq16100;size=5;**	gi|936253934	*Pilumnus hirtellus*	312	100	0	100	8.29E-161	577	483407	Pilumnus hirtellus voucher MT03191 cytochrome oxidase subunit 1 (COI) gene, partial cds; mitochondrial
**Uniq16100;size=5;**	gi|936253812	*Pilumnus hirtellus*	312	100	0	100	8.29E-161	577	483407	Pilumnus hirtellus voucher MT03177 cytochrome oxidase subunit 1 (COI) gene, partial cds; mitochondrial
**Uniq7428;size=12;**	gi|1122285440	*Pilumnus hirtellus*	312	100	0	100	8.29E-161	577	483407	Pilumnus hirtellus isolate K11 cytochrome c oxidase subunit I (COI) gene, partial cds; mitochondrial
**Uniq7428;size=12;**	gi|936254418	*Pilumnus hirtellus*	312	100	0	100	8.29E-161	577	483407	Pilumnus hirtellus voucher MT04581 cytochrome oxidase subunit 1 (COI) gene, partial cds; mitochondrial
**Uniq7428;size=12;**	gi|936254044	*Pilumnus hirtellus*	312	100	0	100	8.29E-161	577	483407	Pilumnus hirtellus voucher MT03176 cytochrome oxidase

										subunit 1 (COI) gene, partial cds; mitochondrial
**Uniq7428;size=12;**	gi|936253934	*Pilumnus hirtellus*	312	100	0	100	8.29E-161	577	483407	Pilumnus hirtellus voucher MT03191 cytochrome oxidase subunit 1 (COI) gene, partial cds; mitochondrial
**Uniq7428;size=12;**	gi|367057081	*Pilumnus hirtellus*	312	100	0	100	8.29E-161	577	483407	Pilumnus hirtellus voucher JSDAz135 cytochrome oxidase subunit 1 (COI) gene, partial cds; mitochondrial
**Uniq25;size=13275;**	gi|1203234130	*Polysiphonia brodiei*	312	100	0	100	8.29E-161	577	159751	Polysiphonia brodiei isolate PD516 cytochrome oxidase subunit 1 (cox1) gene, complete cds; mitochondrial
**Uniq25;size=13275;**	gi|692335831	*Polysiphonia brodiei*	297	100	0	95	1.81E-152	549	159751	Polysiphonia brodiei voucher RMAR1752 cytochrome oxidase subunit 1 (COI) gene, partial cds; mitochondrial
**Uniq25;size=13275;**	gi|692335829	*Polysiphonia brodiei*	289	100	0	92	5.06E-148	534	159751	Polysiphonia brodiei voucher RMAR1869 cytochrome oxidase subunit 1 (COI) gene, partial cds; mitochondrial
**Uniq25;size=13275;**	gi|692335823	*Polysiphonia brodiei*	284	100	0	91	3.05E-145	525	159751	Polysiphonia brodiei voucher RMAR1738 cytochrome oxidase subunit 1 (COI) gene, partial cds; mitochondrial
**Uniq25;size=13275;**	gi|692335827	*Polysiphonia brodiei*	258	100	0	82	8.65E-131	477	159751	Polysiphonia brodiei voucher RMAR1870 cytochrome oxidase subunit 1 (COI) gene, partial cds; mitochondrial
**Uniq3381;size=26;**	gi|1203234198	*Polysiphonia denudata*	265	98.113	0	84	2.42E-126	462	159752	Polysiphonia denudata cytochrome oxidase subunit 1 (cox1) gene, partial cds; mitochondrial
**Uniq3687;size=24;**	gi|1203234198	*Polysiphonia denudata*	265	98.113	0	84	2.42E-126	462	159752	Polysiphonia denudata cytochrome oxidase subunit 1

										(cox1) gene, partial cds; mitochondrial
Uniq3144;size=27;	gi|318085388	*Polysiphonia morrowii*	299	90.97	0	95	1.49E-108	403	173542	Polysiphonia morrowii voucher NZ04-130 cytochrome c oxidase subunit 1 (COI) gene, partial cds; mitochondrial
Uniq3671;size=24;	gi|318085388	*Polysiphonia morrowii*	299	90.97	0	95	1.49E-108	403	173542	Polysiphonia morrowii voucher NZ04-130 cytochrome c oxidase subunit 1 (COI) gene, partial cds; mitochondrial
Uniq39;size=4509;	gi|378554603	*Porphyra umbilicalis*	312	100	0	100	8.29E-161	577	2786	Porphyra umbilicalis mitochondrion, complete genome
Uniq39;size=4509;	gi|343887871	*Porphyra umbilicalis*	312	100	0	100	8.29E-161	577	2786	Porphyra umbilicalis voucher GWS002655 cytochrome oxidase subunit 1 (COI) gene, partial cds; mitochondrial
Uniq39;size=4509;	gi|343887831	*Porphyra umbilicalis*	312	100	0	100	8.29E-161	577	2786	Porphyra umbilicalis voucher GWS007866 cytochrome oxidase subunit 1 (COI) gene, partial cds; mitochondrial
Uniq39;size=4509;	gi|312608226	*Porphyra umbilicalis*	312	100	0	100	8.29E-161	577	2786	Porphyra umbilicalis voucher GWS005951 cytochrome oxidase subunit 1 (COI) gene, partial cds; mitochondrial
Uniq39;size=4509;	gi|343887883	*Porphyra umbilicalis*	312	99.679	0	100	1.07E-159	573	2786	Porphyra umbilicalis voucher GWS008070 cytochrome oxidase subunit 1 (COI) gene, partial cds; mitochondrial
Uniq42;size=4251;	gi|378554603	*Porphyra umbilicalis*	312	100	0	100	8.29E-161	577	2786	Porphyra umbilicalis mitochondrion, complete genome
Uniq42;size=4251;	gi|343887871	*Porphyra umbilicalis*	312	100	0	100	8.29E-161	577	2786	Porphyra umbilicalis voucher GWS002655 cytochrome oxidase subunit 1 (COI) gene, partial cds; mitochondrial
Uniq42;size=4251;	gi|343887831	*Porphyra umbilicalis*	312	100	0	100	8.29E-161	577	2786	Porphyra umbilicalis voucher GWS007866 cytochrome oxidase

										subunit 1 (COI) gene, partial cds; mitochondrial
Uniq42;size=4251;	gi|312608226	*Porphyra umbilicalis*	312	100	0	100	8.29E-161	577	2786	Porphyra umbilicalis voucher GWS005951 cytochrome oxidase subunit 1 (COI) gene, partial cds; mitochondrial
Uniq42;size=4251;	gi|343887883	*Porphyra umbilicalis*	312	99.679	0	100	1.07E-159	573	2786	Porphyra umbilicalis voucher GWS008070 cytochrome oxidase subunit 1 (COI) gene, partial cds; mitochondrial
Uniq3819;size=23;	gi|1141997811	*Ruditapes philippinarum*	312	100	0	100	8.29E-161	577	129788	Ruditapes philippinarum haplotype Hap_46 cytochrome oxidase subunit I gene, partial cds; mitochondrial
Uniq3819;size=23;	gi|1141997807	*Ruditapes philippinarum*	312	100	0	100	8.29E-161	577	129788	Ruditapes philippinarum haplotype Hap_44 cytochrome oxidase subunit I gene, partial cds; mitochondrial
Uniq3819;size=23;	gi|1141997803	*Ruditapes philippinarum*	312	100	0	100	8.29E-161	577	129788	Ruditapes philippinarum haplotype Hap_42 cytochrome oxidase subunit I gene, partial cds; mitochondrial
Uniq3819;size=23;	gi|1141997751	*Ruditapes philippinarum*	312	100	0	100	8.29E-161	577	129788	Ruditapes philippinarum haplotype Hap_16 cytochrome oxidase subunit I gene, partial cds; mitochondrial
Uniq3819;size=23;	gi|874508332	*Ruditapes philippinarum*	312	100	0	100	8.29E-161	577	129788	Ruditapes philippinarum mitochondrion, complete genome
Uniq6949;size=13;	gi|1141997811	*Ruditapes philippinarum*	312	100	0	100	8.38E-161	577	129788	Ruditapes philippinarum haplotype Hap_46 cytochrome oxidase subunit I gene, partial cds; mitochondrial
Uniq6949;size=13;	gi|1141997807	*Ruditapes philippinarum*	312	100	0	100	8.38E-161	577	129788	Ruditapes philippinarum haplotype Hap_44 cytochrome oxidase subunit I gene, partial cds; mitochondrial

Uniq6949;size=13;	gi|1141997803	*Ruditapes philippinarum*	312	100	0	100	8.38E-161	577	129788	Ruditapes philippinarum haplotype Hap_42 cytochrome oxidase subunit I gene, partial cds; mitochondrial
Uniq6949;size=13;	gi|1141997751	*Ruditapes philippinarum*	312	100	0	100	8.38E-161	577	129788	Ruditapes philippinarum haplotype Hap_16 cytochrome oxidase subunit I gene, partial cds; mitochondrial
Uniq6949;size=13;	gi|874508332	*Ruditapes philippinarum*	312	100	0	100	8.38E-161	577	129788	Ruditapes philippinarum mitochondrion, complete genome
Uniq282;size=226;	gi|1122285498	*Watersipora subtorquata*	312	100	0	100	8.29E-161	577	193294	Watersipora subtorquata isolate G3C13 cytochrome c oxidase subunit I (COI) gene, partial cds; mitochondrial
Uniq282;size=226;	gi|90018725	*Watersipora subtorquata*	296	100	0	94	6.50E-152	547	193294	Watersipora subtorquata isolate WsV1 cytochrome oxidase subunit I (COI) gene, partial cds; mitochondrial
Uniq282;size=226;	gi|223861317	*Watersipora subtorquata*	312	97.756	0	100	3.91E-149	538	193294	Watersipora subtorquata mitochondrion, complete genome
Uniq296;size=216;	gi|1122285498	*Watersipora subtorquata*	312	100	0	100	8.29E-161	577	193294	Watersipora subtorquata isolate G3C13 cytochrome c oxidase subunit I (COI) gene, partial cds; mitochondrial
Uniq296;size=216;	gi|90018725	*Watersipora subtorquata*	295	100	0	94	2.34E-151	545	193294	Watersipora subtorquata isolate WsV1 cytochrome oxidase subunit I (COI) gene, partial cds; mitochondrial
Uniq296;size=216;	gi|223861317	*Watersipora subtorquata*	312	97.756	0	100	3.91E-149	538	193294	Watersipora subtorquata mitochondrion, complete genome
Uniq516;size=121;	gi|1122285492	*Watersipora subtorquata*	312	100	0	100	8.29E-161	577	193294	Watersipora subtorquata isolate F2E1 cytochrome c oxidase subunit I (COI) gene, partial cds; mitochondrial
Uniq516;size=121;	gi|342360257	*Watersipora subtorquata*	312	100	0	100	8.29E-161	577	193294	Watersipora subtorquata isolate WsubTB01 cytochrome c oxidase

										subunit 1 (COI) gene, partial cds; mitochondrial
**Uniq516;size=121;**	gi|342360255	*Watersipora subtorquata*	312	100	0	100	8.29E-161	577	193294	Watersipora subtorquata isolate Bry8Wsub cytochrome c oxidase subunit 1 (COI) gene, partial cds; mitochondrial
**Uniq516;size=121;**	gi|237930312	*Watersipora subtorquata*	312	100	0	100	8.29E-161	577	193294	Watersipora subtorquata haplotype Ws1 cytochrome c oxidase subunit 1 (COI) gene, partial cds; mitochondrial
**Uniq516;size=121;**	gi|90018717	*Watersipora subtorquata*	312	99.679	0	100	1.07E-159	573	193294	Watersipora subtorquata isolate WsSI2 cytochrome oxidase subunit I (COI) gene, partial cds; mitochondrial
**Uniq530;size=118;**	gi|1122285492	*Watersipora subtorquata*	312	100	0	100	8.29E-161	577	193294	Watersipora subtorquata isolate F2E1 cytochrome c oxidase subunit I (COI) gene, partial cds; mitochondrial
**Uniq530;size=118;**	gi|342360257	*Watersipora subtorquata*	312	100	0	100	8.29E-161	577	193294	Watersipora subtorquata isolate WsubTB01 cytochrome c oxidase subunit 1 (COI) gene, partial cds; mitochondrial
**Uniq530;size=118;**	gi|342360255	*Watersipora subtorquata*	312	100	0	100	8.29E-161	577	193294	Watersipora subtorquata isolate Bry8Wsub cytochrome c oxidase subunit 1 (COI) gene, partial cds; mitochondrial
**Uniq530;size=118;**	gi|237930312	*Watersipora subtorquata*	312	100	0	100	8.29E-161	577	193294	Watersipora subtorquata haplotype Ws1 cytochrome c oxidase subunit 1 (COI) gene, partial cds; mitochondrial
**Uniq530;size=118;**	gi|90018717	*Watersipora subtorquata*	312	99.679	0	100	1.07E-159	573	193294	Watersipora subtorquata isolate WsSI2 cytochrome oxidase subunit I (COI) gene, partial cds; mitochondrial

Supplementary Figure S1. Sampling–month based rarefaction curves of Chao 1 for both painted and bare panels and for each of the four gene markers. Dashed lines indicate 95% confidence interval.

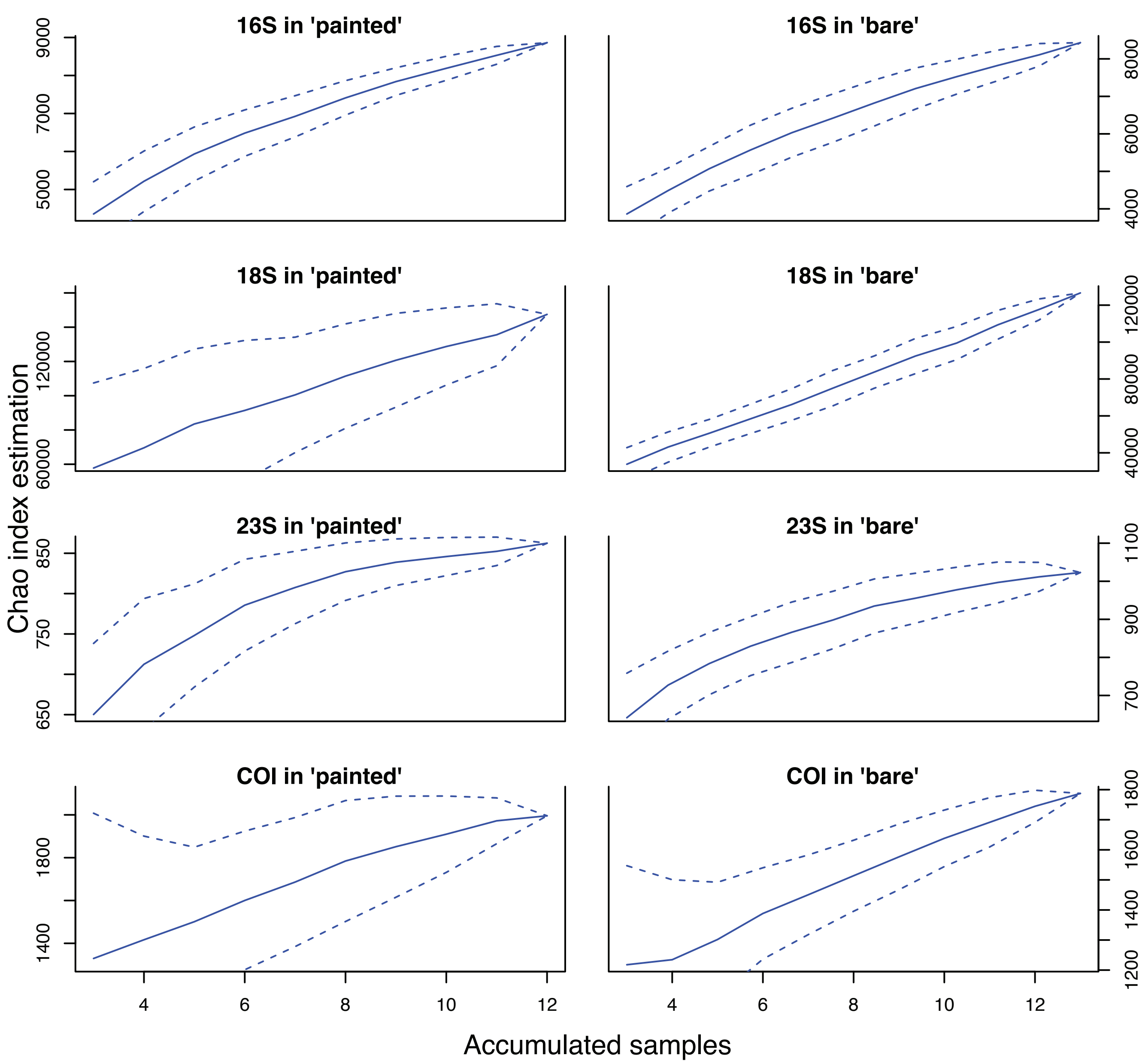

Supplementary File S1. Nucleotide sequences of OTUs found in the COI-derived dataset and with correspondence in at least one of the four NIS databases Inspected.

>Uniq7;size=27913;

TCTTTCAGCAGCTCAAGCTCATTCAGGCCCTTCAGTTGATATGGCAATATTTAGTTTACACTGCGCAGGAGCATCATC
CATTATGGGAGCAATTAATTTTATTACTACAATTATTAATATGAGAGCTCCTGGAATGTCTTTCGACAAACTACCTCTT
TTCGTTTGATCTGTTTTTATCACAGCTTTCTTGTTATTATTATCTCTACCAGTACTAGCAGGAGCAATTACTATGCTTCT
AACTGATAGAAACTTTAACACCACTTTCTTTGACCCAGCTGGTGGGGGAGATCCGGTTTTATATCAACACTTATTT

>Uniq8;size=27711;

AAATAAGTGTTGATATAAAACCGGATCTCCCCCACCAGCTGGGTCAAAGAAAGTGGTGTTAAAGTTTCTATCAGTTA
GAAGCATAGTAATTGCTCCTGCTAGTACTGGTAGAGATAATAATAACAAGAAAGCTGTGATAAAAACAGATCAAAC
GAAAAGAGGTAGTTTGTCGAAAGACATTCCAGGAGCTCTCATATTAATAATTGTAGTAATAAAATTAATTGCTCCCA
TAATGGATGATGCTCCTGCGCAGTGTAAACTAAATATTGCCATATCAACTGAAGGGCCTGAATGAGCTTGAGCTGCT
GAAAGA

>Uniq25;size=13275;

TTTAAGTTCAATTCAAAGTCATTCAGGAGCTGCAGTTGATTTGGCAATTTTTAGCCTTCATTTGTCAGGTGCTTCTTCT
ATTTTAGGAGCTATTAATTTTATTTCTACAATTTTAAATATGCGGAACCCAGGCCAAACATTTTACAGAATTCCTCTTT
TTGTATGATCTATTTTTGTAACAGCTTTTTTACTTTTATTAGCAGTACCTGTTCTTGCAGGAGCTATAACTATGTTATTA
ACTGATCGAAATTTTAACACAGCGTTTTTTGATCCTGCAGGTGGCGGTGATCCTGTACTTTACCAACATTTATTT

>Uniq39;size=4509;

GAATAAATGTTGATATAAAACGGGGTCACCTCCACCTGAAGGATCAAAAAATGTGGTATTAAAATTTCTATCCGTGA
GTAACATTGTAATAGCACCCGCTAATACAGGAACGGCTAATAACAACAGAAAAGCTGTAATTAAGATAGACCAGAC
AAATAATGGAATACGGTACATACTTTGCCCTGGATTGCGCATATTGAATATGGTAGTAATAAAATTAATAGCACCAA
GTATAGAAGAAGCTCCCGATAAATGCAAACTAAAAATAGCTAGATCAACGGCTCCTCCAGAGTGACTTTGAATAGA
ACTTAGT

>Uniq42;size=4251;

ACTAAGTTCTATTCAAAGTCACTCTGGAGGAGCCGTTGATCTAGCTATTTTTAGTTTGCATTTATCGGGAGCTTCTTC
TATACTTGGTGCTATTAATTTTATTACTACCATATTCAATATGCGCAATCCAGGGCAAAGTATGTACCGTATTCCATTA
TTTGTCTGGTCTATCTTAATTACAGCTTTTCTGTTGTTATTAGCCGTTCCTGTATTAGCGGGTGCTATTACAATGTTAC
TCACGGATAGAAATTTTAATACCACATTTTTTGATCCTTCAGGTGGAGGTGACCCCGTTTTATATCAACATTTATTC

>Uniq79;size=1063;

AAAGAGGTGTTGAAATAAAATAGGATCACCACCCCCTGCAGGGTCAAAAAATGACGTATTGAAGTTACGATCAGTC
AAAAGCATAGTAATAGCTCCAGCTAGTACTGGCAAGGACAATAACAGTAAAAAAGCTGTAATTAAGACGGATCAAA
CAAATAAGGGTAATTTGTCTCAAGTTACACCTGGGGCACGCATATTAAAAATTGTTGTAATGAAATTCATAGCGCCT
AAAATAGAGGAGGCACCTGCTAAATGGAGGCTGAAAATTCCCATATCTACGGCACCACCTGAATGGGCTTGTATTC
CAGAAAGA

>Uniq84;size=1010;

TCTTTCTGGAATACAAGCCCATTCAGGTGGTGCCGTAGATATGGGAATTTTCAGCCTCCATTTAGCAGGTGCCTCCT
CTATTTTAGGCGCTATGAATTTCATTACAACAATTTTTAATATGCGTGCCCCAGGTGTAACTTGAGACAAATTACCCT
TATTTGTTTGATCCGTCTTAATTACAGCTTTTTTACTGTTATTGTCCTTGCCAGTACTAGCTGGAGCTATTACTATGCT
TTTGACTGATCGTAACTTCAATACGTCATTTTTTGACCCTGCAGGGGGTGGTGATCCTATTTTATTTCAACACCTCTTT

>Uniq86;size=966;

TTTATCAGGACCTCAAACACATTCAGGAGGATCAGTAGATATGGCAATTTTTAGTTTACATTGTGCAGGTGCTTCATC
TATTATGGGTGCCATAAATTTTATTACAACAATATTTAATATGAGAGCCCCTGGCTTAACTTTAGATAAATTACCATTA

TTTGTTTGGTCAGTCTTAATTACTGCCTTTTTATTACTTTTATCTTTACCTGTATTAGCTGGAGCTATTACGATGTTACT
TACAGATAGAAATTTTAATACAACATTCTTTGATCCAGCAGGTGGAGGTGATCCAGTATTATACCAACATTTATTT

>Uniq87;size=949;

AAATAAATGTTGGTATAATACTGGATCACCTCCACCTGCTGGATCAAAGAATGTTGTATTAAAATTTCTATCTGTAAG
TAACATCGTAATAGCTCCAGCTAATACAGGTAAAGATAAAAGTAATAAAAAGGCAGTAATTAAGACTGACCAAACA
AATAATGGTAATTTATCTAAAGTTAAGCCAGGGGCTCTCATATTAAATATTGTTGTAATAAAATTTATGGCACCCATA
ATAGATGAAGCACCTGCACAATGTAAACTAAAAATTGCCATATCTACTGATCCTCCTGAATGTGTTTGAGGTCCTGA
TAAA

>Uniq106;size=801;

AAATAAATGTTGGTAAAGAATTGGATCTCCTCCACCTGTAGGGTCAAAAAAGGAGGTATTTAAATTTCGATCTGTAA
GTAATATTGTAATTGCTCCGGCCAACACAGGTAATGAAAGGAGTAAAAGAATAACTGTAATAAATACGCTTCACAC
GAAAAGAGGGAGTCGATCGAATGTAAGAGTTTCAGCTCGTATATTAATAACCGTTGATATAAAATTAATGGCCCCTA
AAATTGAGGAAGCTCCAGCTAAATGTAAAGAGAAAATAGATAAATCAACTGAAGCACCTGAGTGAGCGATATTTCT
AGAAAGA

>Uniq127;size=719;

TCTTTCTAGAAATATCGCTCACTCAGGTGCTTCAGTTGATTTATCTATTTTCTCTTTACATTTAGCTGGAGCTTCCTCA
ATTTTAGGGGCCATTAATTTTATATCAACGGTTATTAATATACGAGCTGAAACTCTTACATTCGATCGACTCCCTCTTT
TCGTGTGAAGCGTATTTATTACAGTTATTCTTTTACTCCTTTCATTACCTGTGTTGGCCGGAGCAATTACAATATTACT
TACAGATCGAAATTTAAATACCTCCTTTTTTGACCCTACAGGTGGAGGAGATCCAATTCTTTACCAACATTTATTT

>Uniq135;size=692;

TTTATCAAGTATACAAGCTCACTCCGGGGGATCGGTTGATATGGCAATTTTTAGTCTTCATTTAGCCGGGATATCTTC
TATATTGGGAGCAATGAATTTCATTACAACCATTATAAATATGAGAGCGCCAGGTATTACAATGGACCGAATGCCAT
TATTTGTGTGATCTGTTTTAGTAACTGCCATTTTATTATTGTTATCTTTACCGGTATTGGCCGGGGCTATAACAATGCT
TTTGACAGATCGAAATTTCAATACTGCGTTCTTTGATCCAGCGGGGGGTGGGGACCCAATTTTATATCAACACCTTTT
C

>Uniq139;size=676;

GAAAAGGTGTTGATATAAAATTGGGTCCCCACCCCCCGCTGGATCAAAGAACGCAGTATTGAAATTTCGATCTGTCA
AAAGCATTGTTATAGCCCCGGCCAATACCGGTAAAGATAACAATAATAAAATGGCAGTTACTAAAACAGATCACACA
AATAATGGCATTCGGTCCATTGTAATACCTGGCGCTCTCATATTTATAATGGTTGTAATGAAATTCATTGCTCCCAAT
ATAGAAGATATCCCGGCTAAATGAAGACTAAAAATTGCCATATCAACCGATCCCCCGGAGTGAGCTTGTATACTTGA
TAAA

>Uniq141;size=668;

CCTTTCTGGAATACAAGCCCACTCAGGTGGTGCCGTAGATATGGGAATTTTTAGCCTCCATTTAGCAGGTGCCTCCT
CTATTTTAGGCGCTATGAATTTCATTACAACAATTTTTAATATGCGTGCCCCAGGTGTAACTTGAGACAAACTACCCT
TATTTGTTTGATCCGTCTTAATTACAGCTTTCTTACTGTTATTGTCCTTACCAGTGCTAGCGGGAGCTATTACTATGCT
TTTGACTGATCGTAACTTCAATACGTCATTTTTTGATCCTGCAGGGGGTGGCGATCCTATTTTATTTCAACACCTCTTT

>Uniq161;size=472;

AAATAAATGTTGATATAAAACAGGATCTCCACCTCCTGATGGATCAAAAAATGAAGTATTAAAATTACGATCAGTTA
ATAACATAGTAATAGCTCCAGCTAAAACAGGTACAGCTAATAATAATAAAAATGCAGTTACAAATATAGATCAAACA
AATAAAGGTATTCTATATAAATTTTGTCCCGGATTTCGCATATTTAAAATTGTAGAAATAAAATTTATAGCACCTAATA
TTGAAGAAGCTCCTGATAAATGTAAACTAAAAATAGCTAAATCTACTGAGGCTCCTGAATGACTTTGAATTGAACTT
AAA

>Uniq172;size=448;

TTTAAGTTCAATTCAAAGTCATTCAGGAGCCTCAGTAGATTTAGCTATTTTTAGTTTACATTTATCAGGAGCTTCTTCA
ATATTAGGTGCTATAAATTTTATTTCTACAATTTTAAATATGCGAAATCCGGGACAAAATTTATATAGAATACCTTTAT
TTGTTTGATCTATATTTGTAACTGCATTTTTATTATTATTAGCTGTACCTGTTTTAGCTGGAGCTATTACTATGTTATTA
ACTGATCGTAATTTTAATACTTCATTTTTTGATCCATCAGGAGGTGGAGATCCTGTTTTATATCAACATTTATTT

>Uniq188;size=384;

AAATAAATGTTGGTACAATACAGGATCACCCCCACCAGCAGGATCAAAAAAAGTAGTGTTAAAATTCCTATCAGTTA
AAAGCATGGTGATACCTCCAGCTAAAACAGGAAGAGACAATAGAAGTAAAAAAGCTGTTATTAAAACAGACCAAAC
AAAAAGAGGTAACCTATCCATAGTCATACCAGGAGCACGCATATTAAAAATAGTAGTAATAAAATTAATAGCACCTA
AAATAGAAGCTGCTCCAGATAAGTGTAAACTAAAAATAGCTAAATCAACGGAAGGACCTGAGTGAGCTTGAATACC
ACTTAAG

>Uniq191;size=375;

CTTAAGTGGTATTCAAGCTCACTCAGGTCCTTCCGTTGATTTAGCTATTTTTAGTTTACACTTATCTGGAGCAGCTTCT
ATTTTAGGTGCTATTAATTTTATTACTACTATTTTTAATATGCGTGCTCCTGGTATGACTATGGATAGGTTACCTCTTT
TTGTTTGGTCTGTTTTAATAACAGCTTTTTTACTTCTATTGTCTCTTCCTGTTTTAGCTGGAGGTATCACCATGCTTTTA
ACTGATAGGAATTTTAACACTACTTTTTTTGATCCTGCTGGTGGGGGTGATCCTGTATTGTACCAACATTTATTT

>Uniq199;size=360;

TTTATCCAGCATCCAAACGCATTCAGGAGGGGCAGTTGACATGGCCATTTTTAGTCTTCATTTAGCAGGTGCTTCTTC
TATTTTAGGGGCAATGAATTTTATAACAACTATATTTAATATGCGGGCTCCAGGAATGACAATGGACAGACTCCCAT
TATTTGTATGGTCTATTTTAATCACTGCCTTTTTATTGTTGCTCTCCTTACCTGTATTAGCTGGTGCTATTACTATGCTT
TTAACAGATAGAAATTTTAATACAACTTTCTTTGATCCAGCTGGGGGTGGAGACCCTATTTTATTCCAACATTTATTT

>Uniq211;size=328;

AAATAAATGTTGGAATAAAATAGGGTCTCCACCCCCAGCTGGATCAAAGAAAGTTGTATTAAAATTTCTATCTGTTA
AAAGCATAGTAATAGCACCAGCTAATACAGGTAAGGAGAGCAACAATAAAAAGGCAGTGATTAAAATAGACCATAC
AAATAATGGGAGTCTGTCCATTGTCATTCCTGGAGCCCGCATATTAAATATAGTTGTTATAAAATTCATTGCCCCTAA
AATAGAAGAAGCACCTGCTAAATGAAGACTAAAAATGGCCATGTCAACTGCCCCTCCTGAATGCGTTTGGATGCTG
GATAAA

>Uniq246;size=265;

TCTGTCTAGAAATATTGCTCACTCAGGAGCGTCAGTAGATCTCTCTATTTTTTCTCTACATTTGGCTGGGGCTTCATC
AATTTTAGGAGCTATTAATTTTATATCAACAGTAATTAATATACGAGCTGAGACGCTAACGTTTGATCGTTTACCTTT
ATTCGTTTGAAGAGTATTTATTACAGTAATTCTATTACTTTTATCTCTTCCTGTTCTAGCTGGAGCAATTACAATGTTA
TTAACAGATCGAAATTTAAATACTTCGTTCTTTGACCCTACGGGGGGAGGGGACCCAATTCTTTACCAACACCTATTT

>Uniq279;size=227;

AAATAGGTGTTGGTAAAGAATTGGGTCCCCTCCCCCCGTAGGGTCAAAGAACGAAGTATTTAAATTTCGATCTGTTA
ATAACATTGTAATTGCTCCAGCTAGAACAGGAAGAGATAAAAGTAATAGAATTACTGTAATAAATACTCTTCAAACG
AATAAAGGTAAACGATCAAACGTTAGCGTCTCAGCTCGTATATTAATTACTGTTGATATAAAATTAATAGCTCCTAAA
ATTGATGAAGCCCCAGCCAAATGTAGAGAAAAAATAGAGAGATCTACTGACGCTCCTGAGTGAGCAATATTTCTAG
ACAGA

>Uniq282;size=226;

AAATAAATGTTGGTATAAAATAGGATCTCCCCCACCTGCTGGGTCAAAGAAGGAAGAGTTAAAATTCCGGTCGGTT
AAAAGTATAGTAATAGCCCCTGCTAGTACTGGTAATGATAAAAGTAATAATAGTGCAGTAATAAATACGGCTCAAAC
AAATAATGGGAGGTTTATAAAAGATATTCTTTTTCCTCGTATATTGATAATCGTTACTATAAAATTAATTGCCCCTAAA
ATAGAGGATACCCCAGCAAGATGTAGCGCAAAGATTGCCATATCTACTGAAGATCCTCTATGTGAGATATTAGAGG
ATAGA

>Uniq296;size=216;

TCTATCCTCTAATATCTCACATAGAGGATCTTCAGTAGATATGGCAATCTTTGCGCTACATCTTGCTGGGGTATCCTC
TATTTTAGGGGCAATTAATTTTATAGTAACGATTATCAATATACGAGGAAAAAGAATATCTTTTATAAACCTCCCATT
ATTTGTTTGAGCCGTATTTATTACTGCACTATTATTACTTTTATCATTACCAGTACTAGCAGGGGCTATTACTATACTT
TTAACCGACCGGAATTTTAACTCTTCCTTCTTTGACCCAGCAGGTGGGGGAGATCCTATTTTATACCAACATTTATTT

>Uniq480;size=130;

TCTATCAAGTAATATTGCACATTCTGGTGCTTCTGTAGATTTATCTATTTTTTCTTTACACTTAGCCGGGGCATCTTCA
ATTTTAGGAGCAATTAATTTTATATCAACAGTAATTAATATACGATCTGAAACTCTTACTTTTGACCGACTTCCTTTAT
TTGTTTGAAGTGTATTTATTACCGTAATTTTACTTCTTTTATCATTACCTGTTTTAGCTGGTGCTATTACAATATTATTA
ACTGACCGTAACTTAAACACTTCTTTTTTTGACCCTACTGGTGGGGGAGACCCAATTTTATATCAACATTTATTC

>Uniq512;size=122;

GAATAAATGTTGATATAAAATTGGGTCTCCCCCACCAGTAGGGTCAAAAAAAGAAGTGTTTAAGTTACGGTCAGTTA
ATAATATTGTAATAGCACCAGCTAAAACAGGTAATGATAAAAGAAGTAAAATTACGGTAATAAATACACTTCAAACA
AATAAAGGAAGTCGGTCAAAAGTAAGAGTTTCAGATCGTATATTAATTACTGTTGATATAAAATTAATTGCTCCTAA
AATTGAAGATGCCCCGGCTAAGTGTAAAGAAAAAATAGATAAATCTACAGAAGCACCAGAATGTGCAATATTACTT
GATAGA

>Uniq516;size=121;

CCTATCCTCTAATATCTCTCATAGAGGATCTTCAGTAGATATAGCAATCTTTGCGCTACACTTAGCTGGAGTATCCTC
TATTTTAGGAGCAATTAATTTTATAGTAACAATTATCAATATGCGAGGGAAAAGAATATCTTTTATAAACCTACCATT
ATTTGTTTGAGCCGTATTTATTACTGCACTATTGTTACTTTTATCATTACCAGTACTAGCAGGGGCTATTACTATACTT
TTAACCGACCGGAACTTTAACTCTTCCTTCTTTGACCCAGCAGGAGGAGGGGATCCTATTTTATACCAACATTTATTT

>Uniq530;size=118;

AAATAAATGTTGGTATAAAATAGGATCCCCTCCTCCTGCTGGGTCAAAGAAGGAAGAGTTAAAGTTCCGGTCGGTT
AAAAGTATAGTAATAGCCCCTGCTAGTACTGGTAATGATAAAAGTAACAATAGTGCAGTAATAAATACGGCTCAAAC
AAATAATGGTAGGTTTATAAAAGATATTCTTTTCCCTCGCATATTGATAATTGTTACTATAAAATTAATTGCTCCTAAA
ATAGAGGATACTCCAGCTAAGTGTAGCGCAAAGATTGCTATATCTACTGAAGATCCTCTATGAGAGATATTAGAGG
ATAGG

>Uniq537;size=116;

TTTAAGCTCAATACAAAGTCATTCTGGAGCATCTGTAGATTTAGCTATTTTTAGTTTGCATTTATCAGGAGCATCTTC
GATATTAGGAGCTATAAATTTTATTTCTACAATTTTAAATATGCGAAATCCTGGCCAAAGTTTATATAGAATTCCTTTA
TTTGTATGATCTATATTTGTAACTGCTTTTTTATTATTATTAGCTGTACCTGTTTTAGCAGGAGCTATTACAATGTTACT
GACTGATCGTAATTTTAATACTTCATTTTTTGATCCATCAGGTGGTGGGGATCCTATTTTATATCAACATTTATTT

>Uniq681;size=96;

AAATAAATGTTGATATAAAATAGGATCCCCACCACCTGATGGATCAAAAAATGAAGTATTAAAATTACGATCAGTCA
GTAACATTGTAATAGCTCCTGCTAAAACAGGTACAGCTAATAATAATAAAAAAGCAGTTACAAATATAGATCATACA
AATAAAGGAATTCTATATAAACTTTGGCCAGGATTTCGCATATTTAAAATTGTAGAAATAAAATTTATAGCTCCTAAT
ATCGAAGATGCTCCTGATAAATGCAAACTAAAAATAGCTAAATCTACAGATGCTCCAGAATGACTTTGTATTGAGCT
TAAA

>Uniq13537;size=6;

TTTATCTAGTATTCAAACACATTCTGGGGGATCTGTGGATATGGCGATATTTAGTCTTCATTTAGCGGGAATATCTTC
TATATTGGGTGCTATGAACTTCATAACAACAATTTTTAATATGAGGGCACCGGGGATAACTATGGATAGAATGCCTT
TATTTGTTTGATCTGTTTTAGTAACTGCTTTTTTATTATTATTATCATTGCCAGTATTAGCTGGTAGTATAACAATGCTT
TTAACCGATCGAAACTTTAATACTACTTTTTTTGATCCGGCTGGAGGGGGAGATCCAATATTATATCAACATTTATTT

>Uniq13879;size=6;

AAATAAATGTTGAAAAAGAACAGGGTCACCCCCACCAGCCGGATCGAAAAACCTGGTATTAAGATTACGATCAGTC
AACAGCATGGTAATAGCACCCGCTAACACGGGAAGAGAGAGCAGTAGTAATAAAGCAGTAATAAACACTGACCAA
GTAAAAAGAGAGACCCGAACGAATGTCATAGAATAACTACGCATATTAACAACCGTAGTCATAAAATTAATAGCCCC
AAGAATAGAAGAAACACCTGCTAAGTGAAGAGAAAAAATAGCTATATCAACAGATGAGCCCCTATGAGCAATGTTA
GAGGCTAAT

>Uniq16100;size=5;

GAATAAATGTTGATAAAGCACCGGGTCACCTCCTCCGGCAGGATCAAAGAAAGAGGTATTCAGATTACGGTCTGTA
AGAAGCATAGTAATAGCTCCGGCTAGTACCGGTAAAGATAAAAGAAGTAGAATTGCTGTGATGAATACAGCCCATA
CAAAAAGCGGTATTTGATCTATTGATATACCGAAGGATCGTATATTAATTACGGTAGTTATAAAGTTAACTGCTCCTA
AGATGGAAGAAACCCCTGCCAAATGAAGAGAAAAGATTCCCATATCTACTGAAGCACCTGCATGAGCAATAGCCGC
AGCTAAA

>Uniq1420;size=54;

AAATAAATGTTGGTAAAGAATAGGATCACCACCACCTGACGGATCAAAAAATGAAGTATTAAAATTCCTATCAGTCA
GTAACATTGTAATTGCCCCAGCTAGAACGGGTACTGCTAGTAAAAGTAAAACAGCTGTTACAAAAATTGATCATACA
AAAAGCGGGATCCGATACATACTTTGGCCTGGGTTACGCATATTAAGAATTGTCGATATGAAGTTAACCGCTCCTAA
AATTGAAGAGGCACCTGAAATGTGTAAGCTGAAGATAGCTAGATCGACAGCTGCACCCGAGTGGCTTTGGATTGA
GCTTAAA

>Uniq2061;size=40;

AAAGAGGTGTTGAAATAAAATAGGATCGCCACCCCCTGCAGGATCAAAAAATGACGTATTGAAGTTACGATCAGTC
AAAAGCATAGTAATAGCTCCCGCTAGCACTGGTAAGGACAATAACAGTAAGAAAGCTGTAATTAAGACGGATCAAA
CAAATAAGGGTAGTTTGTCTCAAGTTACACCTGGGGCACGCATATTAAAAATTGTTGTAATGAAATTCATAGCGCCT
AAAATAGAGGAGGCACCTGCTAAATGGAGGCTAAAAATTCCCATATCTACGGCACCACCTGAATGGGCTTGTATTC
CAGAAGGG

>Uniq2123;size=39;

CCTGGCTGGTTCTATCGCCCACAGAGGACCATCTGTAGACCTAGCCATTTTTTCACTTCACCTTGCTGGTGCCTCCTC
TATTCTGGGGTCTTTAAACTTCCTAACCACTGCCTATAACATACGACCAGAAACTCTCATGGCCGAACGAATCCCACT
ATTTGTTTGGTCCCTAATCGTAACTGCCCTACTACTAGTCCTATCACTCCCAGTGTTAGCCGGAGCAATCACAATACT
ATTAACAGATCGTAACCTAAACTCGTCATTCTTTATCCCAGAAGGAGGAGGAGACCCCGTGCTCTACCAACACCTAT
TC

>Uniq2314;size=36;

TTTAAGCTCAATCCAAAGCCACTCGGGTGCAGCTGTCGATCTAGCTATCTTCAGCTTACACATTTCAGGTGCCTCTTC
AATTTTAGGAGCGGTTAACTTCATATCGACAATTCTTAATATGCGTAACCCAGGCCAAAGTATGTATCGGATCCCGC
TTTTTGTATGATCAATTTTTGTAACAGCTGTTTTACTTTTACTAGCAGTACCCGTTCTAGCTGGGGCAATTACAATGTT
ACTGACTGATAGGAATTTTAATACTTCATTTTTTGATCCGTCAGGTGGTGGTGATCCTATTCTTTACCAACATTTATTT

>Uniq3144;size=27;

ACTTAGTTCGATTCAAAGTCATTCTGGAGCTTCAGTTGATCTTGCAATATTTAGTTTACATCTTTCAGGAGCTTCCTCT
ATTTTAGGTGCGATAAATTTTATTTCCACTATTATTAATATGCGTAATCCTGGACAAACTTTTTACAGAATACCTCTTT
TTGTTTGATCTATTTTTGTAACAGCTTTTTTATTATTGTTGGCGGTTCCAGTTCTAGCTGGAGCAATAACTATGTTATT
GACAGATAGAAATTTTAATACTTCATTTTTTGATCCTGCAGGAGGAGGTGATCCTATTTTATATCAGCATTTATTT

>Uniq3381;size=26;

AAATAAATGCTGATATAATACAGGGTCACCTCCACCTGCAGGATCAAAAAAAGAAGTATTAAAATTTCGATCAGTTA
ATAACATTGTAATAGCTCCTGCAAGAACAGGTACTGCTAGTAAAAGTAAAAAAGCTGTCACAAAAATAGATCATACG
AAAAGAGGAATTCTATAAAATGTTTGTCCAGGATTACGCATATTTAAAATTGTTGAAATAAAATTAATAGCTCCTAAA

ATAGAAGAAGCTCCTGACAAGTGTAAGCTAAAAATTGCTAAATCAACTGCTGCTCCTGAATGACTTTGAATTGAACT
TAAG

>Uniq3671;size=24;

AAATAAATGCTGATATAAAATAGGATCACCTCCTCCTGCAGGATCAAAAAATGAAGTATTAAAATTTCTATCTGTCAA
TAACATAGTTATTGCTCCAGCTAGAACTGGAACCGCCAACAATAATAAAAAAGCTGTTACAAAAATAGATCAAACAA
AAAGAGGTATTCTGTAAAAAGTTTGTCCAGGATTACGCATATTAATAATAGTGGAAATAAAATTTATCGCACCTAAA
ATAGAGGAAGCTCCTGAAAGATGTAAACTAAATATTGCAAGATCAACTGAAGCTCCAGAATGACTTTGAATCGAAC
TAAGT

>Uniq3687;size=24;

CTTAAGTTCAATTCAAAGTCATTCAGGAGCAGCAGTTGATTTAGCAATTTTTAGCTTACACTTGTCAGGAGCTTCTTC
TATTTTAGGAGCTATTAATTTTATTTCAACAATTTTAAATATGCGTAATCCTGGACAAACATTTTATAGAATTCCTCTTT
TCGTATGATCTATTTTTGTGACAGCTTTTTTACTTTTACTAGCAGTACCTGTTCTTGCAGGAGCTATTACAATGTTATT
AACTGATCGAAATTTTAATACTTCTTTTTTTGATCCTGCAGGTGGAGGTGACCCTGTATTATATCAGCATTTATTT

>Uniq3819;size=23;

AAATAAATGAATAAATAAAATAGGGTCTCCCAACCCTATTGGATCAAAAAATGTTGTATTAAAATGACGATCACTAA
GCAGCATAGTTAAACCCCCTGCCAAAACTGGTATAGCTACAATCAAAAGAAAACCTGTTACTCCTAAACACCAAGCT
AACATACTACTACGCAAAATTAACATGACACCTGTTCGCATCAATGATGTAGTAATTACAAAATTAATAGAAGCTAA
AATTGAAGAGACACCACCTACGTGAAGAGAAAAAATTACATAATCCACAGCACAACCTGAATGGAACCCAATTGAC
GACAAC

>Uniq3833;size=23;

TTTATCAAGTATACAATTTCACTCAGGGGGATCGGTAGATATGGCAATTTTTAGCCTTCATTTAGCTGGGATATCTTC
AATATTGGGAGCTATGAATTTTATTACAACCATTATAAATATGAGAGCACCAGGGATTACAATGGACCGAATGCCTT
TATTTGTGTGATCTGTTTTAGTAACAGCCGTTTTGTTATTGTTATCTTTACCAGTATTTGCAGGAGCTATAACAATGCT
TTTAACAGATCGAAACTTTAATACTGCATTCTTTGATCCGGCGGGGGGAGGTGATCCTATTTTATATCAACACCTTTT
C

>Uniq4519;size=20;

AAATAAGTGTTGATAGAGAACAGGATCACCGCCTCCTGCAGGGTCAAAAAAAGTAGTATTAAAATTTCTATCGGTTA
AAAGCATAGTTATTGCTCCAGCTAATACAGGAAGGGATAAAAGAAGAAGAATTGCAGTAATTAAAACTGATCAGAC
AAATAAAGGTAATTTATCAAAAGTAATTCCAGGTGCTCTCATATTGAAGATAGTAGTAATAAAATTAATAGCCCCTG
CTATAGAAGAAAAACCAGCGCAATGGAGACTAAATATAGCCATGTCAACCGAACCTCCAGAGTGAGTAATTGGGCC
CGATAAG

>Uniq4695;size=19;

GAATAGATGTTGATATAAAATAGGATCTCCACCACCAGCCGGATCAAAGAAAGAAGTATTAAGGTTTCGATCTGTA
AGTAATATTGTAATAGCCCCAGCAAGAACTGGAAGAGATAATAGAAGAAGAATAACGGTTAGTAGAACTGCTCATA
CAAATAATGGTAGTCGTTCTAGTCGTATTCCGTTTCATCGCATATTAATTGTTGTTGTAATAAAGTTAACTGCCCCTA
AAATAGATGAAGCTCCTGCTAAGTGAAGAGAGAAAATTGCTATATCTACTGATGGTCCCGCATGGGCAAGATTTCTT
GATAGT

>Uniq5051;size=18;

CCTGAGTTCTTATATTTTTCACGGAGGCGCATCTGTGGACTTTACAATTTTTAGACTCCACTTAGCTGGAGTTTCTTC
GCTTCTAGGGGCAGTGAACTTTATCAGAACTGTTTTAAATTTACGGGCATTAGGGATGTTAATAGATCGTATACCTT
TGTTCCCGTGAGCGGTGTTTATTACTGCTATTTTGCTCCTTTTGTCCCTCCCTGTTTTAGCAGGCGCCATCACTATACT
ATTAACAGACCGGAATTTAAACACGTCTTTCTATGACCCTATAGGTGGGGGGGACCCCGTCCTTTACCAGCACTTAT
TT

>Uniq5262;size=17;

AAATAAATGCTGATACAATACTGGATCACCACCACCAGCTGGATCAAAAAAGGTAGTATTAAAATTTCTATCAGTTA
AAAGCATTGTAATACCTCCAGCCAAAACTGGGAGAGATAACAGAAGCAGAAATGCTGTAATTAATACCGACCAAAC
AAAAAGAGGTAATCTATCCATAGTCATACCAGGAGCACGCATATTAAAGATAGTAGTAATAAAATTAATAGCTCCTA
AAATAGACGCCGCTCCAGATAAATGTAAACTAAAGATTGCTAAATCAACGGAAGGACCTGAGTGGGCTTGAATACC
ACTTAGT

>Uniq6230;size=14;

CCTTAGAAGATCAATTGCCCACAGAGGTGGGGCAGTTGATTTAGCTATTTTCTCCCTCCATCTAGCAGGTGCATCCT
CAATTCTAGGGGCAATTAATTTTATTTCTACAATTATTAATATACGATCAGCTAATATATTTATAAGACGAATACCTTT
ATTTGTGTGATCAGTTTTTATCACGGCAATCTTACTTCTCTTATCACTCCCAGTACTTGCAGGGGCTATCACTATATTA
CTCACTGACCGGAATATTAACACTTCTTTTTTTGATCCTTTAGGGGGGGGTGATCCTATTCTTTACCAGCATCTTTTC

>Uniq6328;size=14;

GAAAAGATGCTGGTAAAGAATAGGATCACCCCCCCCTAAAGGATCAAAAAAAGAAGTGTTAATATTCCGGTCAGTG
AGTAATATAGTGATAGCCCCTGCAAGTACTGGGAGTGATAAGAGAAGTAAGATTGCCGTGATAAAAACTGATCACA
CAAATAAAGGTATTCGTCTTATAAATATATTAGCTGATCGTATATTAATAATTGTAGAAATAAAATTAATTGCCCCTA
GAATTGAGGATGCACCTGCTAGATGGAGGGAGAAAATAGCTAAATCAACTGCCCCACCTCTGTGGGCAATTGATCT
TCTAAGG

>Uniq6503;size=14;

TTTGTCTAGTATTCAAACTCATTCTGGGGGATCTGTGGACATGGTGATATTTAGTCTTCATTTAGCAGGAATATCTTC
TATATTGGGTGCTATGAACTTTATAACAACAATTTTTAATATGAGGGCACCGGGGATAACTATGGATAGAATGCCTT
TATTTGTTTGATCTGTTTTAGTAACTGCTTTTTTATTATTGTTATCATTGCCAGTATTAGCCGGTGCTATAACAATGCTT
TTAACCGATCGAAATTTTAATACTACTTTTTTTGATCCGGCTGGAGGGGGGGATCCAATATTATATCAACATTTATTT

>Uniq6528;size=14;

ACTATCAAGAAATCTTGCCCATGCGGGACCATCAGTAGATATAGCAATTTTCTCTCTTCACTTAGCAGGAGCTTCATC
TATTTTAGGGGCAGTTAACTTTATTACAACAACAATTAATATGCGATGAAACGGAATACGACTAGAACGACTACCAT
TATTTGTATGAGCAGTTCTACTAACCGTTATTCTTCTTCTATTATCTCTTCCAGTTCTTGCTGGGGCTATTACAATATTA
CTTACAGATCGAAACCTTAATACTTCTTTCTTTGATCCGGCTGGTGGTGGAGATCCTATTTTATATCAACATCTATTC

>Uniq6949;size=13;

GTTGTCGTCAATTGGGTTCCATTCAGGTTGTGCTGTGGATTATGTAATTTTTTCTCTTCACGTAGGTGGTGTCTCTTC
AATTTTAGCTTCTATTAATTTTGTAATTACTACATCATTGATGCGAACAGGTGTCATGTTAATTTTGCGTAGTAGTATG
TTAGCTTGGTGTTTAGGAGTAACAGGTTTTCTTTTGATTGTAGCTATACCAGTTTTGGCAGGGGGTTTAACTATGCT
GCTTAGTGATCGTCATTTTAATACAACATTTTTTGATCCAATAGGGTTGGGAGACCCTATTTTATTTATTCATTTATTT

>Uniq6993;size=13;

GAAAAGGTGTTGATATAAAATAGGATCACCTCCCCCCGCCGGATCAAAGAATGCAGTATTAAAGTTTCGATCTGTTA
AAAGCATTGTTATAGCTCCTGCAAATACTGGTAAAGATAACAATAACAAAACGGCTGTTACTAAAACAGATCACACA
AATAAAGGCATTCGGTCCATTGTAATCCCTGGTGCTCTCATATTTATAATGGTTGTAATAAAATTCATAGCTCCCAAT
ATTGAAGATATCCCAGCTAAATGAAGGCTAAAAATTGCCATATCTACCGATCCCCCTGAGTGAAATTGTATACTTGA
TAAA

>Uniq7168;size=12;

CTTATCGGGCCCAATTACTCACTCTGGAGGTTCGGTTGACATGGCTATATTTAGTCTCCATTGCGCTGGTTTTTCTTC
TATAGCAGGGGCTATTAATTTTATTACTACTATCTTCAATATGAGAGCACCTGGAATTACTTTTGATAAATTACCTTTA
TTTGTCTGATCAGTTTTAATTACTGCAATTCTTCTTCTTTTATCCCTTCCTGTATTAGCTGGAGCAATAACTATGCTTTT
AACCGATAGAAATTTTAATACTACTTTTTTTGACCCTGCAGGAGGCGGTGATCCTGTTCTCTATCAACACTTATTT

>Uniq7265;size=12;

AAATAAATGTTGATATAATATTGGATCTCCCCCTCCAGCCGGATCAAAAAAAGTAGTATTAAAGTTTCGATCGGTTA
AAAGCATTGTTATACTACCAGCTAATACTGGCAATGATAATAATAATAAAAAAGCAGTTACTAAAACAGATCAAACA
AATAAAGGCATTCTATCCATAGTTATCCCCGGTGCCCTCATATTAAAAATTGTTGTTATGAAGTTCATAGCACCCAAT
ATAGAAGATATTCCCGCTAAATGAAGACTAAATATCGCCATATCCACAGATCCCCCAGAATGTGTTTGAATACTAGA
TAAA

>Uniq7428;size=12;

TTTAGCTGCGGCTATTGCTCATGCAGGTGCTTCAGTAGATATGGGAATCTTTTCTCTTCATTTGGCAGGGGTTTCTTC
CATCTTAGGAGCAGTTAACTTTATAACTACCGTAATTAATATACGATCCTTCGGTATATCAATAGATCAAATACCGCT
TTTTGTATGGGCTGTATTCATCACAGCAATTCTACTTCTTTTATCTTTACCGGTACTAGCCGGAGCTATTACTATGCTT
CTTACAGACCGTAATCTGAATACCTCTTTCTTTGATCCTGCCGGAGGAGGTGACCCGGTGCTTTATCAACATTTATTC

>Uniq10583;size=8;

AAATAAATGTTGATATAATATTGGATCCCCCCCTCCAGCCGGATCAAAAAAAGTAGTATTAAAATTTCGATCGGTTA
AAAGCATTGTTATAGCACCGGCTAATACTGGCAATGATAACAATAATAAAAAAGCAGTTACTAAAACAGATCAAACA
AATAAAGGCATTCTATCCATAGTTATCCCCGGTGCCCTCATATTAAAAATTGTTGTTATAAAGTTCATAGCACCCAAT
ATAGAAGATATTCCTGCTAAATGAAGACTAAATATCACCATGTCCACAGATCCCCCAGAATGAGTTTGAATACTAGA
CAAA

>Uniq11718;size=7;

ACTAAGTGGTATTCAAGCCCACTCAGGTCCTTCCGTTGATTTAGCAATCTTTAGTTTACATTTATCTGGAGCGGCGTC
TATTTTAGGAGCTATTAATTTTATTACTACTATCTTTAATATGCGTGCTCCTGGTATGACTATGGATAGATTACCTCTT
TTTGTTTGGTCGGTATTAATTACAGCATTTCTGCTTCTGTTATCTCTCCCAGTTTTGGCTGGAGGTATTACAATGCTTT
TAACTGATAGAAATTTTAATACTACCTTTTTTGATCCAGCTGGTGGTGGTGATCCAGTATTGTATCAGCATTTATTT
